# Supplementary material for: On the conditions for shifts in metabolic strategies
Source: PLoS Comput Biol. 2026 Jun 26;22(6):e1014417. doi: 10.1371/journal.pcbi.1014417 (PMC13336470; doi:10.1371/journal.pcbi.1014417)
Supplement: S1 Appendix — Supplementary sections 1–18 with glossary, mathematical derivations, and additional background information on the core model and its adaptation to S. cerevisiae. (PDF) [file pcbi.1014417.s001.pdf]

S1 Appendix:
On the conditions for shifts in metabolic strategies

Maarten J. Droste[1,2], Robert Planqué[1], Frank J. Bruggeman[2]

[1]Department of Mathematics, Amsterdam Center for Dynamics and Computation, Vrije Universiteit Amsterdam, Amsterdam, 1081 HV, the Netherlands
[2]Systems Biology Lab, A-LIFE, AIMMS, Vrije Universiteit Amsterdam, Amsterdam, 1081 HZ, the Netherlands

1 Glossary

C_j: Protein costs associated with process j.
D_c: Critical dilution rate after which yeast shifts from respiration to ethanol fermentation in glucose-limited chemostats.
e_j: Concentration of the enzyme catalysing the lumped reaction that represents process j.
e_{NG}: The concentration of growth-unassociated protein that changes with growth rate.
e_Q: The concentration of growth-unassociated protein that is used for maintenance and other 'household' processes, assumed fixed across conditions.
e_{tot}: The total concentration of protein in a cell, assumed fixed across conditions.
f_j: Saturation factor of the enzyme catalysing the lumped reaction that represents process j.
$\langle f(\lambda)\rangle_j$: Saturation function that describes the mean enzyme saturation factor of the individual enzymatic reactions included in lumped reaction j as a function of the growth rate in yeast.
k_j: Catalytic rate constant of the lumped reaction that represents process j.
$\langle k\rangle_j$: The mean catalytic rate constant of the individual enzymatic reactions included in lumped reaction j.
k_A^c: Critical catalytic rate constant of the assimilation pathway during carbon uptake-limited growth.
m_B: The amount of ATP required to synthesise one mole biomass.
m_j: ATP yield per mole of the carbon source, for reactions $j = R, F, G$.
N_j: Number of individual enzymatic reactions included in the sector represented by lumped reaction j.
q: Specific uptake or excretion rate of a compound per gram of dryweight biomass per hour.
S: Carbon source.
v_j: The rate of the lumped reaction that represents process j.
v_A^c: Critical assimilation rate during carbon uptake-limited growth.
$\mathbf{v}_F$: EFM corresponding to the carbon-inefficient (F) mode.
$\mathbf{v}_R$: EFM corresponding to the carbon-efficient (R) mode.
X: Carbon intermediate.
$Y_{B/S}$: Biomass yield per mole carbon source.
$Y_{X/B}$: The amount of carbon intermediate consumed to synthesise one mole of biomass.

α: Convex coefficient associated with an EFM.
ϵ: Proteome efficiency, i.e., the ATP synthesis rate per unit of protein.
λ: Growth rate of a microbial culture.
λ_c: Critical growth rate at which a shift to a different metabolic strategy commences.
λ_c^y: Critical growth rate of yeast during aerobic glucose-limited chemostat cultivation (equal to D_c).
λ_{max}: Maximal growth rate of a microbial culture.
λ_{max}^y: Maximal growth rate of yeast in batch conditions.
λ_{ratio}: The ratio of the maximal growth rate over the critical growth rate.
ϕ_j: Fraction of the total proteome allocated to lumped reaction j.

2 Analytical solution of the core model in the regime $0 < \lambda < \lambda_c$

In the growth rate regime $0 < \lambda < \lambda_c$, the carbon-efficient mode is used in isolation. This means that the carbon-inefficient mode is not used and thus its catabolic protein pool vanishes: $e_F = 0$. The three

steady-state equations (1) and (5) now admit a unique solution in terms of the three remaining protein concentrations. To find this solution, we use the equation $v_j = k_j e_j f_j$ for the reaction rates and $e_F = 0$ to rewrite the steady-state equations as

$$\begin{aligned}\lambda &= \frac{k_B e_B f_B}{e_{tot}} \\ m_R k_R e_R f_R &= m_B k_B e_B f_B \\ k_A e_A f_A &= k_R e_R f_R + Y_{X/B} k_B e_B f_B.\end{aligned} \tag{S1}$$

Solving this linear system for the protein concentrations as function of the growth rate yields

$$\begin{aligned}e_B &= \frac{\lambda e_{tot}}{k_B f_B} \\ e_R &= \frac{m_B k_B f_B}{m_R k_R f_R} e_B = \frac{m_B}{m_R k_R f_R} \lambda e_{tot} \\ e_A &= \frac{k_R f_R}{k_A f_A} e_R + \frac{Y_{X/B} k_B f_B}{k_A f_A} e_B = \frac{\frac{m_B}{m_R} + Y_{X/B}}{k_A f_A} \lambda e_{tot}.\end{aligned} \tag{S2}$$

It is customary to use protein concentrations normalised to the total protein concentration. In terms of these protein fractions $\phi_j = e_j/e_{tot}$, eq. (S2) becomes

$$\phi_B(\lambda) = \frac{1}{k_B f_B}\lambda \equiv C_B \lambda;\ \phi_R(\lambda) = \frac{m_B}{m_R k_R f_R}\lambda \equiv C_R\lambda;\ \phi_A^R(\lambda) = \frac{\frac{m_B}{m_R} + Y_{X/B}}{k_A f_A}\lambda \equiv C_A^R \lambda. \tag{S3}$$

This is the steady-state solution of the core model for the carbon-efficient, R-strategy. The constants C_j collect corresponding parameter combinations and may be interpreted as (lumped) protein costs for each lumped reaction. It should be noted that the saturation of the assimilation pathway (f_A) is in fact not constant, but depends on the growth rate via the Monod relation. Then we find a unique solution for the protein pools only when an extra degree of freedom is added. Since the assimilation fraction is typically small, we expect the effect of a variable f_A to be negligible, so we set it constant.

3 Derivation of the equation for λ_{max}

To determine an equation for λ_{max}, we study the carbon-inefficient mode in isolation. Since $\phi_R = 0$, we solve the steady-state equations (1) and (5) for the remaining protein fractions, similar to the derivation in the previous section. This yields

$$\phi_B(\lambda) = \frac{1}{k_B f_B}\lambda \equiv C_B \lambda; \quad \phi_F(\lambda) = \frac{m_B}{m_F k_F f_F}\lambda \equiv C_F\lambda; \quad \phi_A^F(\lambda) = \frac{\frac{m_B}{m_F} + Y_{X/B}}{k_A f_A}\lambda \equiv C_A^F \lambda. \tag{S4}$$

The solution for the biosynthetic protein fraction ϕ_B matches its counterpart for the carbon-efficient mode (eq. (S3)), demonstrating that biomass synthesis is completely independent of the used catabolic mode in this model.

The growth-associated protein fractions in eq. (S4) are also proportional to the growth rate. The remaining protein fraction is used for growth-unassociated tasks. In other words, when the carbon-inefficient mode is employed in isolation, it has its own growth-unassociated fraction $\phi_Q + \phi_{NG}^F$. This fraction decreases with growth rate because the total protein concentration is fixed. Since e_Q is assumed fixed, this decreasing pool is again e_{NG}^F, with corresponding fraction

$$\phi_{NG}^F(\lambda) = 1 - (\phi_Q + \phi_B(\lambda) + \phi_F(\lambda) + \phi_A^F(\lambda)) = 1 - \phi_Q - \lambda\left(C_B + C_F + C_A^F\right). \tag{S5}$$

Similar to the carbon-efficient mode, the carbon-inefficient mode reaches its maximal growth rate when the growth-unassociated protein sector e_{NG}^F is depleted. Solving eq. (S5) at $\phi_{NG}^F = 0$ for λ gives

$$\lambda_{max} = \frac{1 - \phi_Q}{C_B + C_F + C_A^F}. \tag{S6}$$

When this growth rate is reached, no alternative strategy exists to further increase the growth rate. So, the maximal growth rate of the carbon-inefficient mode is in fact the maximal growth rate of the cell in this model.

4 Analytical solution of the core model in the regime $\lambda_c < \lambda < \lambda_{max}$

The optimisation problem

$$\text{(S7)} \quad \min_{\mathbf{e}} \Big\{ v_A \Big| \mathbf{N}\mathbf{v} = \mathbf{0}, e_A + e_R + e_F + e_B + e_Q \leq e_{tot}, \lambda = \frac{v_B}{e_{tot}} \text{ fixed}, \\ \forall j: v_j = k_j e_j f_j, e_j \geq 0, \{k_j, f_j, e_Q, e_{tot}\} \text{ constant} \Big\},$$

also given in eq. (10), is the formulation of the core model in chemostat conditions, where the growth rate is set by the experimentalist via the dilution rate.

In the regime $0 < \lambda < \lambda_c$, only the carbon-efficient mode is used, so the steady-state equations (1) and (5) admit a unique solution in terms of the protein fractions (eq. (S3)). In the regime $\lambda_c < \lambda < \lambda_{max}$, a shift to the inefficient mode occurs because the NG-sector is depleted. Because the carbon assimilation rate is minimised, also the protein investment in the carbon-inefficient mode is minimised, since this mode requires more carbon due to its lower yield. So, the NG-sector remains empty across the regime $\lambda_c < \lambda < \lambda_{max}$. Again, this eliminates one of the variables and allows us to derive a unique solution to the steady-state equations in this regime, in terms of the protein fractions. We find that the corresponding expressions in terms of the model parameters are cumbersome. We use the expressions for the critical growth rate (19) and the maximal growth rate (22) to reformulate these as

$$\text{(S8)} \quad \begin{aligned} \phi_B(\lambda) &= C_B \lambda \\ \phi_R(\lambda) &= C_R \left(\frac{\lambda_{max} - \lambda}{\lambda_{max}/\lambda_c - 1} \right) \\ \phi_F(\lambda) &= C_F \left(\frac{\lambda - \lambda_c}{1 - \lambda_c/\lambda_{max}} \right) \\ \phi_A(\lambda) &= \frac{\lambda_c \lambda_{max} (C_A^R - C_A^F)}{\lambda_{max} - \lambda_c} + \lambda \left(\frac{\lambda_{max} C_A^F - \lambda_c C_A^R}{\lambda_{max} - \lambda_c} \right). \end{aligned}$$

Here, the constants C_j correspond to the same parameter combinations as in eq. (17) and (21).

The biosynthetic protein fraction ϕ_B satisfies the same linear relation with the growth rate in both regimes. The respiratory fraction $\phi_R(\lambda)$ attains its maximal value at the critical growth rate and vanishes at the maximal growth rate. Conversely, $\phi_F(\lambda)$ vanishes at the critical growth rate and attains its maximal value at the maximal growth rate. These results are summarised in Fig 2A. They agree with experimental findings [1; 2; 3].

In the regime $\lambda_c < \lambda < \lambda_{max}$, the flux vector is given by a convex combination (8) of the two EFMs $\mathbf{v}_R$ and $\mathbf{v}_F$ with nonzero convex coefficients α_R and α_F. Because $\alpha_R(\lambda_c) = 1$ and $\alpha_F(\lambda_{max}) = 1$, we may express the fluxes through the lumped $R-$ and $F-$ reactions as the product of the corresponding convex coefficients and the maximal flux values. This yields

$$\text{(S9)} \quad \begin{aligned} v_R(\lambda) &= \alpha_R(\lambda) v_R(\lambda_c) \\ v_F(\lambda) &= \alpha_F(\lambda) v_F(\lambda_{max}). \end{aligned}$$

Expressions for the convex coefficients then follow directly from eq. (S8) as

$$\text{(S10)} \quad \begin{aligned} \alpha_R(\lambda) &= \frac{v_R(\lambda)}{v_R(\lambda_c)} = \frac{\phi_R(\lambda)}{\phi_R(\lambda_c)} = \frac{\lambda_{max} - \lambda}{\lambda_{max} - \lambda_c} \\ \alpha_F(\lambda) &= \frac{v_F(\lambda)}{v_F(\lambda_{max})} = \frac{\phi_F(\lambda)}{\phi_F(\lambda_{max})} = \frac{\lambda - \lambda_c}{\lambda_{max} - \lambda_c}. \end{aligned}$$

Here, we used that flux ratios are equivalent to protein fraction ratios when saturations are constant. These expressions for the convex coefficients satisfy the required property that $\alpha_R(\lambda) + \alpha_F(\lambda) = 1$.

5 Proteome efficiency definitions

In Section 3.3, we computed the proteome efficiencies for both modes as the ATP synthesis rate per unit of total protein expended to sustain the ATP flux. Using the solutions for the protein fractions (eq. (17), (21)),

these condense to

$$\epsilon_R \equiv \frac{m_R v_R(\lambda)}{e_B(\lambda) + e_R(\lambda) + e_A^R(\lambda)} = \frac{m_B}{C_B + C_R + C_A^R} = \frac{m_B}{1-\phi_Q}\lambda_c$$
$$\epsilon_F \equiv \frac{m_F v_F(\lambda)}{e_B(\lambda) + e_F(\lambda) + e_A^F(\lambda)} = \frac{m_B}{C_B + C_F + C_A^F} = \frac{m_B}{1-\phi_Q}\lambda_{max},$$

If we assume constant enzyme saturations and ATP requirement m_B, the proteome efficiencies are also constant. Furthermore, these expressions show that the proteome efficiencies are directly related to the maximal growth rates: a shift to the inefficient F-mode thus implies its higher (maximal) growth rate as well as its higher proteome efficiency. In fact, taking the ratio of the proteome efficiencies shows that

(S11)
$$\epsilon_{ratio} \equiv \frac{\epsilon_F}{\epsilon_R} = \frac{\lambda_{max}}{\lambda_c} \equiv \lambda_{ratio}.$$

In other words, the break-even analysis in Section 3.5 yields identical results in terms the proteome efficiencies.

The proteome efficiency of the carbon-inefficient F-mode exceeds that of the carbon-efficient R-mode if its protein costs are lower, i.e.,

(S12)
$$C_R + C_A^R > C_F + C_A^F.$$

The biosynthetic protein costs C_B do not appear, because these are the same for both strategies. This idea allows for a different definition for the proteome efficiency. Instead of considering the ATP synthesis rate per total amount of protein, an alternative is to compute the ATP synthesis rate per unit of catabolic protein expended to sustain the ATP flux. This yields the catabolic proteome efficiencies

$$\epsilon_R^{cat} \equiv \frac{m_R v_R(\lambda)}{e_R(\lambda) + e_A^R(\lambda)} = \frac{m_B}{C_R + C_A^R}$$
$$\epsilon_F^{cat} \equiv \frac{m_F v_F(\lambda)}{e_F(\lambda) + e_A^F(\lambda)} = \frac{m_B}{C_F + C_A^F}.$$

These expressions cannot be simplified in terms of the critical and maximal growth rate. Instead, taking their ratio gives the inequality

(S13)
$$\epsilon_{ratio}^{cat} \equiv \frac{\epsilon_F^{cat}}{\epsilon_R^{cat}} = \frac{C_R + C_A^R}{C_F + C_A^F} > \frac{C_B + C_R + C_A^R}{C_B + C_F + C_A^F} = \frac{\lambda_{max}}{\lambda_c} \equiv \lambda_{ratio}.$$

A similar inequality was derived by Basan et al. [4] in their model for overflow metabolism in *E. coli.*

There is no reason to prefer one of the above definitions over the other, as both give a measure of the ATP synthesis rate per unit of protein. Furthermore, the condition $\epsilon_F > \epsilon_R$ is equivalent to $\epsilon_F^{cat} > \epsilon_R^{cat}$, because both are satisfied only when eq. (S12) holds. Regardless of the definition used, the proteome efficiencies do not encode additional information about the metabolic shift that is not already contained in the critical and maximal growth rates.

The various definitions presented here quantify the recent discussion on the concept of proteome efficiency and on the cause of overflow metabolism in yeasts [5; 6]. This will be analysed further by computing the proteome efficiencies in the yeast model in Supplementary Section 17.

6 Break-even analysis

In Section 3.5 we reformulated the break-even condition $\lambda_{ratio} = 1$ in terms of the model parameters as

(S14)
$$\frac{N_R}{m_R k_R f_R} = \frac{N_F}{m_F k_F f_F} + \frac{(m_R - m_F)N_A}{m_R m_F k_A f_A}.$$

Here, we use this relation to derive expressions for the curves depicted in the phase diagrams in Fig 3, that represent the break-even condition in terms of different parameter pairs.

Solving eq. (S14) for the ATP yield of the R-mode gives

(S15)
$$m_R = \frac{m_F k_F f_F (k_R f_R + k_A f_A N_R)}{k_R f_R (k_F f_F + k_A f_A N_F)}.$$

This equation represents the 'break-even point' for the ATP yield of the R-mode. It decreases with the catalytic constant of the R-mode (k_R), as also illustrated by Fig 3B. This means that either one or both

these parameters should be small (compared to their counterpart for the F-mode) in order to observe the metabolic shift. On the other hand, the break-even point for m_R increases linearly with the number of enzymatic reactions N_R with some offset, as depicted in Fig 3C. This is because the protein costs C_R increase with the number of reactions. So, a shift will occur only if the ATP generated per reaction by the carbon-efficient mode is small enough. Similar linear behaviour arises in Fig 3E when analysing eq. (S15) as function of m_F. The break-even point for m_R decreases with the number of reactions N_F, as presented in Fig 3F. Because increasing length N_F increases the protein costs C_F, this also requires a lower ATP yield for the R-mode for a shift to occur. This relation is useful, as fermentation pathways may differ in length in various organisms. For example, *Clostridium pasteurianum* can ferment glucose to lactate or butyrate [7]. The latter involves six enzymatic reactions more.

We also solve eq. (S14) for the catalytic constant of the R-mode, giving its break-even point

$$k_R = \frac{m_F k_F f_F k_A f_A N_R}{f_R f_F k_F (m_R - m_F) + k_A f_A m_R f_R N_F}. \tag{S16}$$

The break-even point for k_R increases hyperbolically with the catalytic constant of the F-mode, as depicted in Fig 3D. This may be interpreted similar to the break-even relation between the ATP yields: when the catalytic constant of the R-mode increases, also that of the F-mode should increase to preserve the shift.

We can also use eq. (S14) to study relations between other parameter pairs that are not presented in Fig 3. For instance, eq. (S15) reveals that $m_R(k_F)$ is a hyperbolically increasing function, as presented in Fig AA. Eq. (S16) and Fig AB show that k_R increases with m_F. For both relations, the interpretation is the same as for the catalytic constants in Fig 3D.

Instead of studying the break-even condition for parameters of the carbon-efficient R-mode, we can also inspect the parameters of the F-mode. Solving eq. (S14) for the ATP yield of the F-mode gives

$$m_F = \frac{m_R k_R f_R (k_F f_F + k_A f_A N_F)}{k_F f_F (k_R f_R + k_A f_A N_R)}. \tag{S17}$$

This break-even point decreases as function of the catalytic constant k_F, while it increases as function of the number of reactions N_F. So, this gives the same behaviour as for the ATP-yield of the R-mode, but then with the shifting and non-shifting regions swapped. These two cases are presented in Fig AC and D. The interpretation of these figures is also reversed with respect to Fig 3A and B, as increasing the protein costs C_F will eliminate the shift. The arrow points in the direction that the regions move if the protein costs of the R-mode increase.

7 Analysis of metabolic shifts as function of nutrient quality during carbon uptake-limited growth

Here, we further analyse the case of uptake-limited growth on varying carbon sources for a constant titrated level of the uptake system. The quality of the different carbon sources may depend on various factors. First, we show that the catalytic rate constant of the assimilation pathway (k_A) is the best measure for the nutrient quality in our model. The concept of nutrient quality was introduced in Scott et al. [8] and recently studied by Mukherjee et al. [9]. Both studies state that the growth rate is proportional to the fraction of catabolic enzyme that catalyses the nutrient influx, in our model given by assimilation fraction (ϕ_A), with the nutrient quality as proportionality constant. Studying the solutions for the assimilation fraction for both modes in eq. (17) and (21) shows that this proportionality constant is given by C_A^{-1}, the inverse of the protein costs for assimilation. The only parameters contained in the protein costs that may differ per carbon source are the catalytic rate constant (k_A) and the carbon required per mole of biomass synthesised ($Y_{X/B}$). The carbon requirement affects only the biosynthetic part of the metabolic network, and therefore does not affect the shift in the catabolic mode. This leaves k_A as the only parameter that varies with carbon source that may affect the shift. This parameter thus represents the nutrient quality in our model.

We note that the concentration of the carbon source may also differ in uptake-limited conditions. This directly sets the saturation factor for assimilation (f_A). A more complete representation of the nutrient quality is therefore the product $k_A f_A$. For simplicity, we assume that the assimilation saturation factor

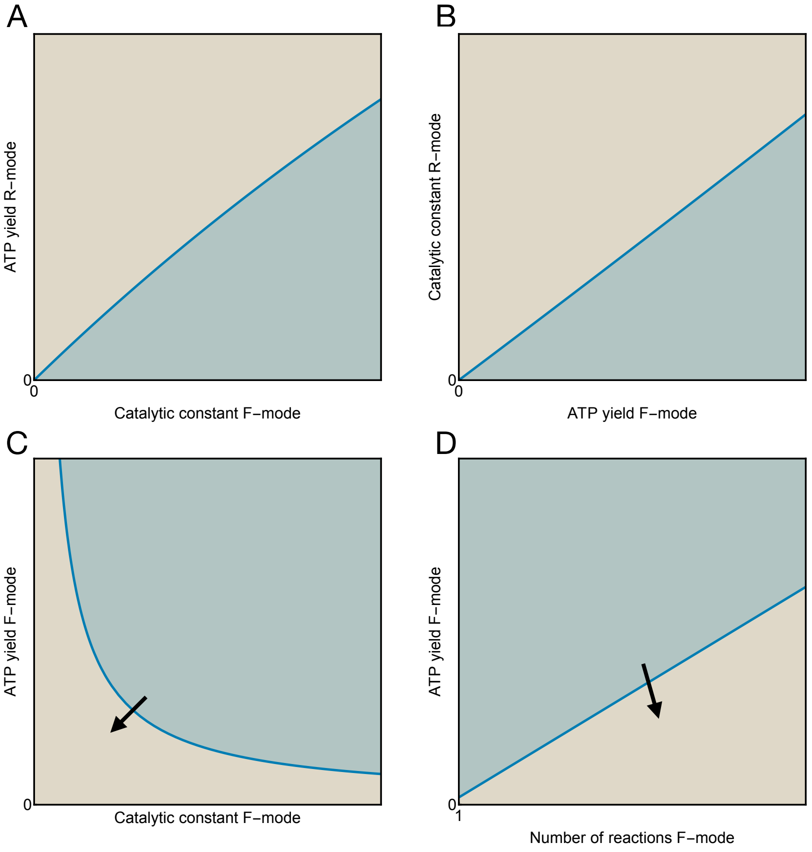

Fig A. **Parameter perturbations in the model affect the occurrence of overflow metabolism.** The break-even analysis in terms of other parameter pairs that are not discussed in Section 3.5. For parameter configurations in the blue region, a metabolic shift occurs. In the brown region a shift will never occur. The arrow in C) and D) indicates the direction in which the break-even condition moves if the protein costs of the carbon-efficient mode increase.

remains fixed across different carbon sources, so we only vary k_A. Relaxing this assumption does not alter the analysis below.

7.1 Analytical solution to the optimisation problem in carbon uptake-limited conditions as function of the nutrient quality

The optimisation problem corresponding to this case of uptake-limited growth follows from adjusting eq. (9) by setting e_A constant and k_A as independent parameter, giving

$$\max_{\mathbf{e}}\left\{\lambda = \frac{v_B}{e_{tot}}\middle|\mathbf{Nv} = \mathbf{0}, e_A + e_R + e_F + e_B + e_Q \leq e_{tot}, k_A \text{ fixed}, \right. \\ \left. \forall j : v_j = k_j e_j f_j, e_j \geq 0, \{k_j, f_j, e_A, e_Q, e_{tot}\} \text{ constant}\right\}. \tag{S18}$$

This adjustment breaks the equivalence with the optimisation problem for chemostat conditions (eq. (10)), and thus requires a separate solution parameterised by k_A, that we will derive here.

Just as the other optimisations, eq. (S18) also maximises the biomass yield. At low nutrient quality, the carbon uptake rate and thus also the growth rate are low. In this regime, enzyme can be invested in growth-unassociated processes, hence the (equality) constraint on e_{tot} in eq. (S18) is not active. As before, only the carbon-efficient mode will be used in this regime. We therefore study this pathway in isolation again, which yields a unique solution to the steady-state equations (1) and (5) in terms of the protein concentrations and the growth rate

$$\begin{aligned} \lambda_R(k_A) &= Y^R_{B/S} f_A \frac{e_A}{e_{tot}} k_A \\ e^R_B(k_A) &= C_B Y^R_{B/S} f_A e_A k_A \\ e_R(k_A) &= C_R Y^R_{B/S} f_A e_A k_A \\ e_F(k_A) &= 0 \\ e_{NG}(k_A) &= e_{tot} - e_Q - e_A \left(1 + (C_B + C_R) Y^R_{B/S} f_A k_A\right). \end{aligned} \tag{S19}$$

Here, we use the parameter combinations C_j and the biomass yield from eq. (12) for notational convenience.

This solution is similar to the solution to eq. (10) in the regime $0 < \lambda < \lambda_c$ in eq. (17). Here, the growth-associated protein concentrations increase linearly with the nutrient quality instead of the growth rate. This diminishes the growth-unassociated pool e_{NG}, until it is depleted at a critical nutrient quality. This has the same interpretation as the critical growth rate. Solving $e_{NG} = 0$ for k_A gives

$$k^c_A = \frac{e_{tot} - e_Q - e_A}{(C_B + C_R) Y^R_{B/S} f_A e_A}. \tag{S20}$$

The maximal nutrient quality k^{max}_A follows from studying the carbon-inefficient mode in isolation, analogous to the derivation in Supplementary Section 3. We do not give the explicit solution in terms of the protein concentrations, as this follows directly from replacing all super- and subscripts R in eq. (S19) by F. We find

$$k^{max}_A = \frac{e_{tot} - e_Q - e_A}{(C_B + C_F) Y^F_{B/S} f_A e_A}. \tag{S21}$$

For growth on a carbon source with maximal quality, the cell thus uses solely the carbon-inefficient mode. Furthermore, $k^{max}_A > k^c_A$ because $C_R > C_F$ and $Y^R_{B/S} > Y^F_{B/S}$. This is equivalent to the condition $\lambda_{max} > \lambda_c$, but reformulated in terms of the nutrient quality.

In the regime $k^c_A < k_A < k^{max}_A$, a convex combination of both modes is used. In this regime, $e_{NG} = 0$, so the optimisation problem (S18) again has an analytical solution for the protein concentrations. Here, we focus on the protein concentrations for the catabolic processes, as this is where the shift occurs. In the regime $k^c_A < k_A < k^{max}_A$, these are given by

$$\begin{aligned} e_R(k_A) &= C_R Y^R_{B/S} f_A e_A \left(\frac{k^{max}_A - k_A}{k^{max}_A / k^c_A - 1}\right) \\ e_F(k_A) &= C_F Y^F_{B/S} f_A e_A \left(\frac{k_A - k^c_A}{1 - k^c_A / k^{max}_A}\right). \end{aligned} \tag{S22}$$

These expressions are similar to their solution (S8) in chemostat conditions. The protein concentration e_R decreases, while e_F increases. This represents the gradual shift from the efficient to the inefficient mode as the nutrient quality increases. The corresponding fluxes are

$$\begin{aligned} v_R(k_A) &= k_R f_R e_R(k_A) = \frac{m_B}{m_R} Y^R_{B/S} f_A e_A \left(\frac{k^{max}_A - k_A}{k^{max}_A / k^c_A - 1}\right) \\ v_F(k_A) &= k_F f_F e_F(k_A) = \frac{m_B}{m_F} Y^F_{B/S} f_A e_A \left(\frac{k_A - k^c_A}{1 - k^c_A / k^{max}_A}\right). \end{aligned} \tag{S23}$$

We obtain the corresponding convex coefficients analogous to eq. (S10), giving

$$\begin{aligned} \alpha_R(k_A) &= \frac{v_R(k_A)}{v_R(k_A^c)} = \frac{e_R(k_A)}{e_R(k_A^c)} = \frac{k_A^{max} - k_A}{k_A^{max} - k_A^c} \\ \alpha_F(k_A) &= \frac{v_F(k_A)}{v_F(k_A^{max})} = \frac{e_F(k_A)}{e_F(k_A^{max})} = \frac{k_A - k_A^c}{k_A^{max} - k_A^c}. \end{aligned} \tag{S24}$$

Similarly, these expressions satisfy $\alpha_R(k_A) + \alpha_F(k_A) = 1$.

7.2 Analytical solution to the optimisation problem in carbon uptake-limited conditions as function of the growth rate

The catalytic constant of the assimilation pathway may be difficult to obtain experimentally. Also, the critical nutrient quality (S20) and maximal nutrient quality (S21) depend significantly on the constant titrated concentration of the assimilation enzyme. Instead, we may express the solution to eq. (S18) in the regime $k_A^c < k_A < k_A^{max}$ in terms of quantities that are easier to obtain experimentally, such as the growth rate λ.

The corresponding critical growth rate is obtained from $\lambda_R(k_A)$ in eq. (S19) by substituting the critical nutrient quality k_A^c. Similarly, the corresponding maximal growth rate follows from substituting the maximal nutrient quality k_A^{max} in $\lambda_F(k_A)$, which is the solution for the growth rate when the inefficient mode is used in isolation. This yields

$$\begin{aligned} \lambda_R(k_A^c) &= \frac{1 - \phi_Q - \phi_A}{C_B + C_R} \\ \lambda_F(k_A^{max}) &= \frac{1 - \phi_Q - \phi_A}{C_B + C_F}. \end{aligned} \tag{S25}$$

These expressions differ from the critical growth rate (19) and the maximal growth rate (22) obtained for chemostat conditions, because the constant assimilation fraction ϕ_A now appears in the numerator. Reformulating the protein concentrations in eq. (S22) in terms of the growth rate gives

$$\begin{aligned} e_R(\lambda) &= C_R e_{tot} \left(\frac{\lambda_F(k_A^{max}) - \lambda}{\lambda_F(k_A^{max})/\lambda_R(k_A^c) - 1} \right) \\ e_F(\lambda) &= C_F e_{tot} \left(\frac{\lambda - \lambda_R(k_A^c)}{1 - \lambda_R(k_A^c)/\lambda_F(k_A^{max})} \right). \end{aligned} \tag{S26}$$

These protein concentrations only differ from their analogous solutions (S8) in chemostat conditions through the different expressions for the critical and maximal growth rate in eq. (S25). So, the general mathematical structure of the solution to the optimisation problems remains conserved across different experimental conditions.

The overflow product flux is proportional to the flux through the F process (v_F). Reformulating the solution for v_F in eq. (S23) as function of the growth rate gives

$$v_F(\lambda) = k_F f_F e_F(\lambda) = \frac{m_B}{m_F} e_{tot} \left(\frac{\lambda - \lambda_R(k_A^c)}{1 - \lambda_R(k_A^c)/\lambda_F(k_A^{max})} \right). \tag{S27}$$

This shows that the overflow flux is a linear function of the growth rate, both for chemostat and carbon uptake-limited batch conditions. Furthermore, both its slope and intersection with the λ-axis are directly determined by the critical and the maximal growth rates. In the limit of small ϕ_A, the expressions for these growth rates in eq. (S25) reduce to the analogous expressions in chemostat conditions, respectively in eq. (19) and (22), for low assimilation costs (C_A). In other words, the overflow line appearing in both chemostat cultures and in uptake-limited batch cultures in different nutrient conditions can be explained using the same principles.

8 Construction of the core model for yeast

We adapt the core model to *S. cerevisiae* cultivated in aerobic glucose-limited chemostats. The corresponding reaction network is depicted in Fig 4. Here, the uptake of carbon is described by a separate transport reaction T. Glycolysis G degrades glucose into pyruvate, thereby gaining m_G ATP per glucose.

Pyruvate is then degraded into byproducts by one of the two catabolic modes. Respiration R degrades pyruvate into carbon dioxide and water, which yields an additional m_R ATP per glucose via the TCA cycle and oxidative phosphorylation. Ethanol fermentation F degrades pyruvate into ethanol. The concentrations for the internal metabolites (ATP, glucose and pyruvate) satisfy the differential equations

$$\begin{aligned} \dot{ATP} &= m_G v_G + m_R v_R - m_B v_B \\ \dot{Glc_{in}} &= v_T - v_G \\ \dot{Pyr} &= v_G - v_F - v_R. \end{aligned} \tag{S28}$$

The equation for pyruvate does not include a pyruvate flux towards biosynthesis, because we neglect carbon required for biosynthesis. A different approach is to assume a constant biosynthetic carbon demand, as modeled by the parameter $Y_{X/B}$ in the general core model. Obtaining this parameter requires a detailed dissection of the carbon- and energy fluxes for the catabolic and anabolic part of the metabolic network. Furthermore, the biosynthetic carbon demand may change when the cell shifts from respiration to fermentation. Methods exist to account for this [10], but this is beyond the scope of this work, because we assume that catabolism and anabolism are independent. We therefore decided to neglect biosynthetic carbon entirely, and adjust for this after evaluating the model. This is discussed in Supplementary Section 12.

The differential equations in eq. (S28) may be reformulated in terms of a flux vector $\mathbf{v_y} = (v_T\, v_G\, v_R\, v_F\, v_B)^T$ and the corresponding stoichiometric matrix

$$\mathbf{N_y} = \begin{pmatrix} 0 & m_G & m_R & 0 & -m_B \\ 1 & -1 & 0 & 0 & 0 \\ 0 & 1 & -1 & -1 & 0 \end{pmatrix}. \tag{S29}$$

We assume balanced growth conditions, where metabolism operates at steady state, i.e., $\mathbf{N_y v_y} = \mathbf{0}$. The two EFMs of the reaction network in Fig 4 correspond to respiration and fermentation. They follow from the stoichiometric matrix as

$$\mathbf{v}_{res} = v_B \begin{pmatrix} \frac{m_B}{m_R+m_G} \\ \frac{m_B}{m_R+m_G} \\ \frac{m_B}{m_R+m_G} \\ 0 \\ 1 \end{pmatrix}, \quad \mathbf{v}_{fer} = v_B \begin{pmatrix} \frac{m_B}{m_G} \\ \frac{m_B}{m_G} \\ 0 \\ \frac{m_B}{m_G} \\ 1 \end{pmatrix}. \tag{S30}$$

Both EFMs have a biomass yield that we compute by taking the ratio of the last over the first entry (following eq. (11)), giving

$$Y_{cat}^{res} = \frac{m_R + m_G}{m_B}, \quad Y_{cat}^{fer} = \frac{m_G}{m_B}. \tag{S31}$$

Because carbon for biosynthesis is not included, these yields describe the biomass yield per mole of glucose that is used by catabolism. The higher biomass yield of respiration directly follows from its higher ATP yield.

The total protein concentration in a yeast cell is assumed fixed [11] and decomposed into the protein sectors as

$$e_{tot} = e_T + e_G + e_R + e_F + e_B + e_{NG} + e_Q. \tag{S32}$$

Next to e_{tot}, we also assume that the growth-unassociated Q-sector and the transporter enzyme have a fixed concentration [3; 8].

We implement the yeast model as an optimisation problem by minimising the carbon transport rate for variable protein concentrations at a fixed growth rate. Because the transporter enzyme concentration is assumed fixed, minimisation of the carbon transport rate reduces to minimisation of the transporter saturation factor. The optimisation problem for the yeast model is then given by

$$\begin{aligned} \min_{\mathbf{e}} \Big\{ f_T \big| \mathbf{N_y v_y} = \mathbf{0}, e_T + e_G + e_R + e_F + e_B + e_{NG} + e_Q = e_{tot}, \lambda = \frac{v_B}{e_{tot}} \text{ fixed}, f_T \geq 0, \\ \forall j: v_j = \langle k \rangle_j \frac{e_j}{N_j} \langle f(\lambda) \rangle_j, e_j \geq 0 \text{ and } \{\langle k \rangle_j, N_j, e_T, e_Q, e_{tot}\} \text{ constant} \Big\}. \end{aligned} \tag{S33}$$

The equations for the lumped reaction rates v_j are derived in the next section. Model parameters and their values are presented in Table F. A Mathematica implementation of the yeast model is provided in the source code on Zenodo.

9 Protocol for reducing GEMs to core models

The core model presented in this work is not tailored to any metabolic shift in a specific organism. Adapting this model to a specific organism requires identification of the essential metabolic pathways and protein sectors that play a significant role during the shift. Each growth-associated sector j is then described by a lumped reaction with flux v_j. These fluxes may be expressed in terms of kinetic parameters of the individual enzymatic reactions that are contained in the sector. These parameters can be obtained from a GEM integrated with proteomics data, thereby calibrating the model with experimental data. Using this top-down approach, we reduce the large metabolic network from the GEM to a core metabolic network, such as the one presented here. Erdrich et al. [12] use another method for automated reduction of GEMs. Their method, however, typically results in a core model that exceeds manageable size for the mathematical analysis conducted in this study.

In this section, we derive expressions for the fluxes v_j in the core model adapted to yeast. This method is straightforward to generalise to other unicellular organisms. We start from the complete metabolic network of yeast that consists of N enzyme-catalysed reactions. The rate of reaction i is given by $v_i = k_i e_i f_i$. We assume that each enzyme e_i belongs to only one of the seven protein sectors. In other words, we may decompose the set of N metabolic reactions as disjoint subsets per sector. Each subset consists of the N_j individual enzymatic reactions that are contained in sector j, such that $N = \sum_j N_j$. The total protein concentration then decomposes as

$$e_{tot} = \sum_{i=1}^{N} e_i = e_T + \sum_{i=1}^{N_G} e_i^G + \sum_{i=1}^{N_R} e_i^R + \sum_{i=1}^{N_F} e_i^F + \sum_{i=1}^{N_B} e_i^B + \sum_{i=1}^{N_{NG}} e_i^{NG} + \sum_{i=1}^{N_Q} e_i^Q. \tag{S34}$$

We assume that there is only one transport reaction. We then define the enzyme concentrations per sector as

$$e_j \equiv \sum_{i=1}^{N_j} e_i^j, \tag{S35}$$

such that we retrieve eq. (S32).

Now, we focus on the growth-associated protein sectors, since we describe only these by a lumped reaction with flux v_j. Due to variations in reaction stoichiometry, flux ratios within sectors are not necessarily equal to one. For instance, each time the upper part of glycolysis runs, the lower part has to run twice to preserve steady state. These flux ratios within sectors we call the *intrasector* stoichiometries. These are different from the *intersector* stoichiometries, which are defined by the entries of the EFMs in eq. (S30).

In principle, the intrasector stoichiometries may be obtained per sector. This is straightforward for linear metabolic pathways such as glycolysis, but it becomes more involved when considering sectors that consist of more complex pathways, such as respiration or biomass synthesis. Furthermore, these intrasector stoichiometries may differ per EFM. Obtaining these stoichiometries is difficult when a combination of EFMs is used, which is the main regime of interest in this study. Therefore, we disregard the intrasector stoichiometries by assuming that all fluxes within a sector are identical, i.e., $v_i^j = v_j$ for all reactions in sector j. Rewriting eq. (S35) by using the rates $v_i = k_i e_i f_i$ and applying this assumption gives

$$e_j = \sum_{i=1}^{N_j} e_i^j = \sum_{i=1}^{N_j} \frac{v_i^j}{k_i^j f_i^j} = v_j \sum_{i=1}^{N_j} \frac{1}{k_i^j f_i^j} = v_j N_j \left\langle \frac{1}{kf} \right\rangle_j. \tag{S36}$$

Here, $\langle ... \rangle_j$ denotes the arithmetic mean over the reactions in sector j. Reformulating this gives an equation for the lumped reaction fluxes

$$v_j = \left\langle \frac{1}{kf} \right\rangle_j^{-1} \frac{e_j}{N_j}. \tag{S37}$$

The ratio $\frac{e_j}{N_j}$ represents the average enzyme concentration per reaction in sector j.

Because we assume that carbon transport consists of only one reaction, its flux trivially reduces to

$$v_T = \left(\frac{1}{k_T f_T}\right)^{-1} e_T = k_T e_T f_T. \tag{S38}$$

For the other growth-associated sectors $j \in \{G, R, F, B\}$ that contain more reactions we derive alternative approximation for the lumped reaction fluxes v_j. First, we assume that the catalytic rate constants k_i and saturations f_i are independent parameters. This means that these parameters do not covary. This results in the approximation

$$v_j^H = \left\langle \frac{1}{k} \right\rangle_j^{-1} \frac{e_j}{N_j} \left\langle \frac{1}{f} \right\rangle_j^{-1} \tag{S39}$$

that expresses the lumped reaction fluxes in terms of the harmonic means of k_i and f_i. Second, we can use the general relation that arithmetic means are always larger than harmonic means. In other words, we have a lower bound $\langle k \rangle_j \geq \left\langle \frac{1}{k} \right\rangle_j^{-1}$, $\langle f \rangle_j \geq \left\langle \frac{1}{f} \right\rangle_j^{-1}$ for the arithmetic means. This gives

$$v_j^A = \langle k \rangle_j \frac{e_j}{N_j} \langle f \rangle_j \geq v_j^H. \tag{S40}$$

This expresses the flux through lumped reaction j as a product of the mean catalytic rate constant, the mean saturation factor and the mean enzyme concentration per reaction in the sector. Therefore, v_j^A should be interpreted as the mean flux through sector j.

The question remains which of these three approximations (S37), (S39) or (S40), is the best to use for the core model, i.e., which of these models the (mean) flux through the sectors most accurately. The first approximation (S37) is not convenient, because this makes use of products of catalytic constants and saturation factors for individual reactions. Catalytic constants and protein concentrations (from which saturations can be computed, as explained in the following section) are, however, typically measured as separate quantities during experiments. Representing them separately allows us to use their individual properties, such as the fact that saturation factors can not exceed one, when calibrating the model with experimental data.

Because of the inequality in eq. (S40), the approximation in terms of harmonic means in eq. (S39) appears to be more accurate. However, the accuracy of these approximations also depends on the intrasector stoichiometries, which are not taken into account. If the average intrasector stoichiometric coefficient exceeds one, the approximation in terms of arithmetic means might be more accurate than the approximation in terms of harmonic means. So, *a priori*, there is no justification to prefer one of these approximations for the lumped reaction fluxes.

Therefore, we should establish a different criterion to select the best approximation for the lumped reaction fluxes in the yeast model. Two key characteristics of overflowing yeast are the critical dilution rate $D_c = 0.275\,\mathrm{h}^{-1}$ in glucose-limited chemostats and the maximal growth rate in glucose batch, which is $\lambda_{max} = 0.47\,\mathrm{h}^{-1}$ for the strain used in Elsemman et al. [3]. The yeast model should be calibrated to these two growth rates. Following the methods of the next section, we computed the harmonic means and arithmetic means of the catalytic rate constants and the enzyme saturations and ran the model in Mathematica for both approximations. Using the arithmetic means results in a critical and maximal growth rate that are in the right order of magnitude, while using the harmonic means is less accurate. Based on this analysis, we decided to use the approximation of the lumped reaction fluxes in eq. (S40) in terms of the arithmetic means. Further calibration of model parameters to find the observed critical and maximal growth rate is discussed in Supplementary Section 11.

10 Saturation functions and catalytic constants in the yeast model

The remaining parameters of the yeast model are determined from proteomics data from Elsemman et al. [3]. These are the mean catalytic rate constants $\langle k \rangle_j$, the mean enzyme saturations $\langle f \rangle_j$ and the number of individual enzymatic reactions N_j per sector.

10.1 Pathway categorisation and number of reactions per sector

First, the metabolic pathways and corresponding enzymatic reactions included in the GEM from Elsemman et al. [3] need to be categorised in terms of the protein sectors used in the yeast model. This categorisation, given in Table A, is based on the COG categories [13]. We only include enzymatic reactions that are given a reaction ID, as these are the reactions that are considered to be metabolic. This results in a set of reactions I_j that contains all enzymatic reactions from the GEM that are associated with the (coarse-grained) sector j. For example, the biomass synthesis sector contains all pathways and corresponding reactions that are related to protein synthesis, because we assume that biomass consists only of proteins.

Intuitively, the number of enzymatic reactions N_j per sector j should correspond to $|I_j|$, the number of enzymatic reactions in each set I_j. However, these reaction sets also contain other involved reactions that do not directly contribute to the net molecular conversion performed by the sector. The core model only describes metabolic processes that are essential to model the metabolic shift. Therefore, it should only take distinct molecular conversions into account. The number of reactions $|I_j|$ in the reaction sets is not an accurate representation of the distinct molecular conversions that should be modeled for these sectors. Instead, these lengths N_j are obtained from standard literature and given in Table B. The number of reactions required for biomass synthesis exceeds that of all other sectors, but it is not exactly known. Therefore, we do not include a value in Table B. Instead, we use this parameter to calibrate the model to experimental data, as is discussed further in Supplementary Section 11.

**Sector in the yeast model**	**Included pathways from [3]**
Transport	Glucose transport
Glycolysis	Glycolysis
Respiration	Mitochondrial carriers, Mitochondrial ribosome, Oxidative phosphorylation, TCA cycle, TIM/TOM translocase
Fermentation	Ethanol fermentation
Biomass synthesis	Amino acid biosynthesis, Amino acid-tRNA ligases, Protein folding, Ribosome, Ribosome assembly factors, Translation elongation factors, Translation initiation factors, tRNA modification

Table A. **Metabolic pathways from the GEM in Elsemman et al. [3], categorised per protein sector in the yeast model.** Biomass synthesis contains all pathways related to protein synthesis, of which the ribosome is a subset.

Although not all reactions in the sets I_j are taken into account to compute the number of distinct conversions N_j, all these reactions do contribute to the mean kinetic constant and mean enzyme saturation of the sectors. All these reactions are therefore included to compute these means. An exception to this is fermentation, for which we only include the two canonical fermentation reactions that are catalysed by the enzymes pyruvate decarboxylase and alcohol dehydrogenase to compute the mean parameters. This manual curation for fermentation was possible, since $|I_F| = 7$. In principle, such a manual check should be carried out for all sectors. However, this is infeasible and will likely not give significantly different results for the other sectors.

10.2 Computing saturations for individual reactions and special cases

Changes in environmental conditions affect intracellular concentrations and thus also enzyme saturations. Some studies explicitly model enzyme kinetics and reactant concentrations to account for changing saturations [14; 15]. However, for coarse-grained models, this usually shows only qualitative agreement between the model and experimental data. A different approach is to infer enzyme saturations from proteomics data. Elsemman et al. [3] report measured protein mass fractions e_i^{exp} from chemostat experiments and GEM-predicted protein mass fractions e_i^{GEM}. We convert these to concentration fractions by dividing the mass fractions by the molar protein mass. The enzyme saturations now follow from assuming that the GEM predicts the right fluxes, i.e., we assume that $v_i^{GEM} = v_i^{exp}$, where v_i^{exp} is the actual flux through reaction i.

Parameter symbol	Included pathways	Number of reactions
N_T	Glucose transport	1
N_G	Glycolysis	10
N_R	Oxidative phosphorylation, TCA cycle, Mitochondrial carriers	20
N_F	Ethanol fermentation	2

Table B. **Table with number of reactions per process in the yeast model.** Because $N_T = 1$, this parameter is not used throughout the rest of this work. In yeast, oxidative phosphorylation is separated into five reaction steps. The TCA cycle consists of the eight standard reactions. The number of reactions related to mitochondrial carriers (7) is obtained from Elsemman et al. [3].

The GEM predicts a minimal enzyme investment (e_i^{GEM}) required to sustain this flux, as it sets all enzyme saturations to one. If we additionally assume that the GEM contains the correct catalytic constants, i.e., $k_i^{GEM} = k_i^{exp}$, then the enzyme saturation of reaction i is

$$f_i^{exp} = \frac{e_i^{GEM}}{e_i^{exp}}. \tag{S41}$$

As emphasised earlier, the number of reactions $|I_j|$ in the reaction set of process j usually does not correspond to the number of distinct molecular conversions N_j. This is partially due to conversions that can not be described by a single reaction catalysed by one enzyme. Such special conversions also require a slightly different computation of the corresponding saturation. In the yeast model, we account for two of these special cases.

The first case concerns multimeric enzymes, which are protein complexes that consist of multiple subunits. Although the complex catalyses the reaction, the subunits appear as separate proteins in the proteomics data. Since the data do not distinguish between free or bound subunits, we assume that all subunits are bound into the catalysing complex. If a subunit with concentration e_{im} appears multiple times in a complex with concentration $e_i^{complex}$, it is assigned a stoichiometric coefficient n_{im}. The subunit with the lowest concentration (after normalising for the stoichiometries) limits formation of the complex. So, the minimum of the individual subunit concentrations gives an approximation for the concentration of the complex

$$e_i^{complex} \approx \min_m \frac{e_{im}}{n_{im}}. \tag{S42}$$

For each multimeric enzyme, this approximation is used to compute its concentration from the data for the individual subunits. This calculation is done for both the experimental data and the GEM predictions from Elsemman et al. [3]. Plugging in these concentrations in eq. (S41) then gives the (approximated) saturation of each multimeric enzyme.

Second, enzymes exist that catalyse the same reaction. These are called iso-enzymes. Because enzyme saturations are determined by the concentrations of substrate and products of the reaction, these iso-enzymes should have the same saturation factor. For reactions catalysed by multiple iso-enzymes, the total concentration of catalysing enzymes (e_i^{iso}) is obtained by summing the concentrations e_{in} of all iso-enzymes n as

$$e_i^{iso} = \sum_n e_{in}. \tag{S43}$$

Again, this is computed for both the experimental data and for the GEM predictions from [3]. Plugging in these concentrations in eq. (S41) gives the saturation of the reaction catalysed by iso-enzymes. Some reactions are catalysed by multiple iso-enzymes that are also multimeric. In that case, both cases need to be accounted for.

10.3 Determination of saturation functions

The proteomics data in Elsemman et al. [3] is obtained from glucose-limited chemostat experiments set at different dilution rates, which we call D_l. The GEM was run at slightly different dilution rates D_k by the

authors. For each D_l, we find the k such that D_k is closest to D_l. Thereby we match the experimentally studied dilution rates to the simulated ones. Using eq. (S41), this results in an array $f_{il} = f_i(D_l)$ for the saturation of each reaction i at dilution rates D_l. For some reactions, experimental data shows expression of the catalysing enzyme, while the GEM predicted its concentration to be zero. These reactions are neglected because their saturations can not be computed from eq. (S41). In other cases, the computed saturation exceeds one. By definition, this is no feasible value for the saturation factor. These cases are likely caused by an underestimation of the catalytic constant k_i^{GEM}. This results in a predicted concentration e_i^{GEM} that exceeds the measured e_i^{exp}, even at maximal predicted enzyme saturation. We adjust these underestimated catalytic constants by increasing them until $f_{il} \leq 1$ for all dilution rate D_l. This results in both feasible saturation values and (more) realistic estimations of the effective catalytic constants. A similar adjustment is used by Battjes et al. [5].

The saturation arrays f_{il} are then grouped per reaction set I_j according to the categorisation in Table A. For each sector j in the yeast model, we then compute the arithmetic mean of the saturation per individual reaction at each dilution rate D_l. This results in arrays with elements

$$\langle f_l \rangle_j = \sum_{i \in I_j}^{|I_j|} \frac{f_{il}}{|I_j|}. \tag{S44}$$

The resulting dataset is provided on Zenodo. We plot this data as function of the growth rate (which equals the dilution rate in the chemostat) and fit functions $\langle f(\lambda) \rangle_j$ to it. These functions we refer to as the saturation functions.

The data $\langle f_l \rangle_j$ together with the fitted saturation functions are depicted in Fig B. The mean saturation data for glycolysis show distinct linear behaviour before and after the critical dilution rate. Therefore, we have fit a piecewise linear function to this data. The saturation of enzymes used for ethanol fermentation is close to zero at low dilution rates, because ethanol is produced only after the critical dilution rate. For higher dilution rates, the saturation increases hyperbolically. This behaviour is also captured by a piecewise continuous function. Fig BC shows that a clear trend is missing in the mean saturation of respiration. Obtaining a saturation function $\langle f(\lambda) \rangle_R$ from this data is problematic. Therefore, we assume that $\langle f(\lambda) \rangle_R$ is a hyperbolically increasing function. This results in a monotonic specific oxygen uptake rate (before and after the critical growth rate) that is consistent with experimental data [3]. A different approach is to use a piecewise continuous function that exhibits a drop around the critical dilution rate. This is a better fit to the saturation data. However, it gives results for the respiratory protein fraction and oxygen uptake rate that fit less well to experimental data. Therefore, we use the hyperbolically increasing form for $\langle f(\lambda) \rangle_R$ in the rest of this work. For the enzymes involved in biomass synthesis, we obtain a linear saturation function $\langle f(\lambda) \rangle_B$ from the data.

10.4 Catalytic rate constants

The GEM in Elsemman et al. [3] makes use of (estimated) catalytic rate constants k_i^{GEM} to compute reaction fluxes. We obtain the mean catalytic rate constants $\langle k \rangle_j$ by computing the arithmetic mean of the catalytic constants per sector as

$$\langle k \rangle_j = \sum_{i \in I_j}^{|I_j|} \frac{k_i^{GEM}}{|I_j|}. \tag{S45}$$

Here, we include the effective k_i^{GEM} that we get by increasing the underestimated catalytic constants so that all saturation values remain below one. We omit proteins for which no value of the catalytic constant is reported. The resulting mean catalytic constants are given in Table C.

For biomass synthesis, we do not use the mean catalytic constant of the reactions in this sector as parameter in the yeast model. This is because we assume that biomass consists only of protein. This means that the biomass synthesis rate v_B represents the protein synthesis rate. The corresponding catalytic constant is the protein translation rate by the ribosome, or more specifically its peptide elongation rate k_{ribo}.

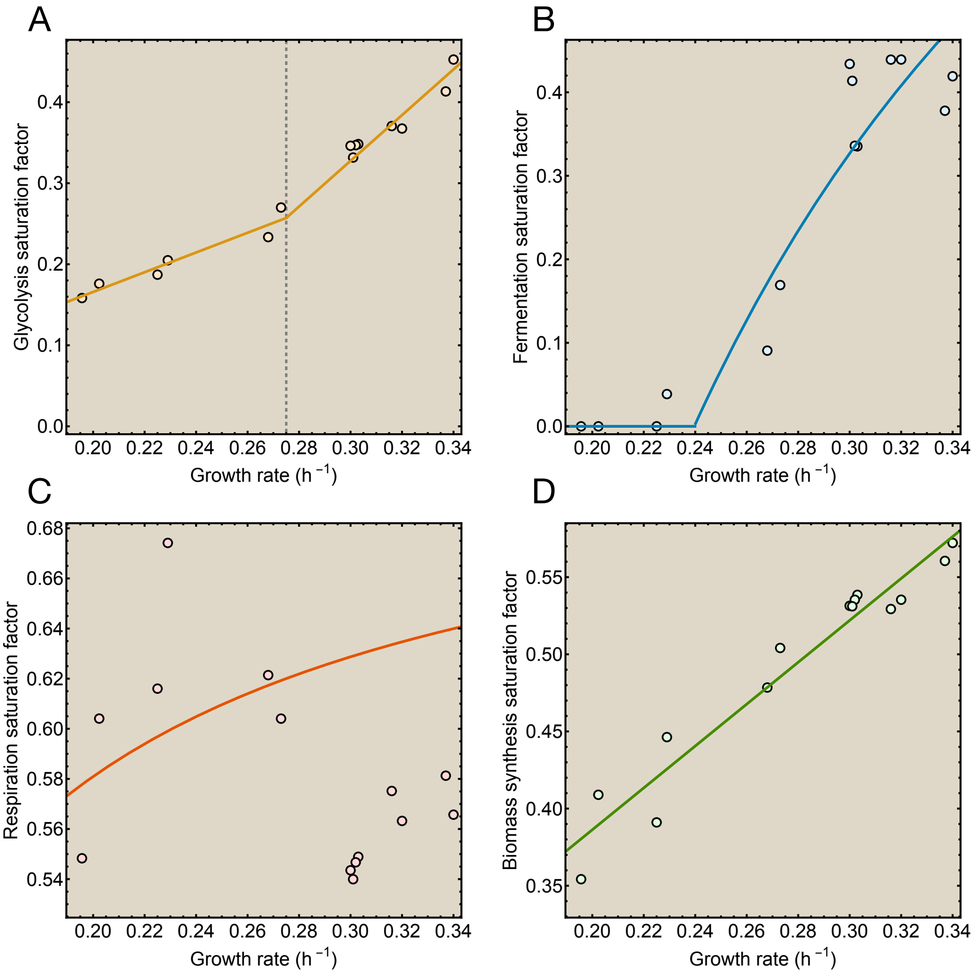

Fig B. **Saturation functions used in the yeast model.** Saturation functions are fit to data obtained from proteomics data, adjusted with permission from [3] (Creative Commons Attribution 4.0 International License). The saturation functions describe the mean saturation factor of the four growth-associated processes in the yeast model. The saturation functions increase monotonically with the growth rate.

Sector in the yeast model	Catalytic constant symbol	Catalytic constant value (h^{-1})
Transport	$\langle k \rangle_T$	$7.2 \cdot 10^5$
Glycolysis	$\langle k \rangle_G$	$1.19 \cdot 10^6$
Respiration	$\langle k \rangle_R$	$4.35 \cdot 10^6$
Fermentation	$\langle k \rangle_F$	$1.68 \cdot 10^6$
Biomass synthesis	k_{ribo}	126

Table C. **Mean catalytic rate constant for each sector in the yeast model.** For k_{ribo}, we use the value reported by Elsemman et al. [3].

11 Calibration of the yeast model

Using the parameter values from the previous sections, we compute the solution to the optimisation problem (S33) for yeast numerically in Mathematica. The results show a shift from respiration to fermentation.

However, the dilution rate when the shift initiates does not correspond to the observed critical dilution rate $D_c = \lambda_c^y = 0.275\,\mathrm{h}^{-1}$ for yeast in aerobic glucose-limited chemostats. Also, the computed maximal growth rate is inconsistent with the observed value $\lambda_{max}^y = 0.47\,\mathrm{h}^{-1}$ for the strain used in Elsemman et al. [3]. These discrepancies may be due to the size of the core model, or the approximation of fluxes in eq. (S40) in terms of arithmetic means of parameters (although this gave more accurate results than using the approximation in eq. (S39) in terms of harmonic means). Finding the observed values for the critical and maximal growth rate in the yeast model therefore requires additional parameter calibration.

Some of the catalytic constants of individual enzymatic reactions were underestimated in the GEM, as discussed in Supplementary Section 10. We increased these cases appropriately to the effective values. These effective values are more realistic, but this method does not guarantee that they are exactly correct. So, this suggests that the (mean) catalytic constants (S45) are parameters with high uncertainty. Therefore, we argue that these are the appropriate parameters to adjust in order to calibrate the model. The catalytic constant of biomass synthesis is set as the peptide elongation rate k_{ribo} and is obtained from the literature, so this parameter is not adjusted. We also use the number of biosynthetic reactions N_B as calibration parameter, since it is not possible to obtain its exact value.

The calibrated values of these parameters are given in Table D. It should be noted that the catalytic constant of respiration requires a 100-fold decrease with respect to its mean value $\langle k \rangle_R$. This likely indicates that $\langle k \rangle_R$ is a poor representation of the mean catalytic constant of respiration. Another explanation may be that respiratory fluxes can not be approximated well in terms of mean enzyme-kinetic parameters.

**Parameter description**	**Parameter symbol**	**Calibrated value**
Catalytic constant of glycolysis	k_G^{cal}	$0.43\ \langle k \rangle_G$
Catalytic constant of respiration	k_R^{cal}	$0.0077\ \langle k \rangle_R$
Catalytic constant of fermentation	k_F^{cal}	$1.1\ \langle k \rangle_F$
Number of biosynthetic reactions	N_B^{cal}	142

Table D. **Calibrated parameters and their values in the yeast model.**

12 Adjustment and results for carbon uptake in the yeast model

In the yeast model, we only account for glucose that is degraded into byproducts by the catabolic modes that charge ATP. However, glucose is also required to synthesise precursors for biomass. This leads to a model prediction for the glucose uptake flux that is significantly below the observed uptake. To compare the results of the yeast model with experimental data, we need to correct for this unaccounted biosynthetic carbon. We do this by adjusting the specific glucose uptake flux predicted by the model, represented by the dashed orange line in Fig CA. We increase the predicted glucose uptake rate such that it matches the experimental data from Elsemman et al. [3] at the critical and maximal growth rate. The resulting adjustment factor is the fraction of imported glucose that is used for catabolic processes ($\frac{S_{cat}}{S_{tot}}$), depicted in Fig CB. This fraction is constant before the critical growth rate, because the flux ratios (and thus yields) are constant when only one EFM is used. The catabolic glucose fraction increases in the regime $\lambda_c < \lambda < \lambda_{max}$. This follows from the lower ATP yield per mole glucose of fermentation. Multiplying the dashed orange line in Fig CA by the inverse of the catabolic glucose fraction ($\frac{S_{tot}}{S_{cat}}$) results in the solid orange line. This represents the total specific glucose uptake flux, that agrees better with the experimental data.

We apply the same adjustment to the biomass yield. First, we convert the yield obtained from the model to units of gram dry weight per gram glucose. This conversion uses the molar weight of an average protein $MW_{prot} = 50$ g/mmol protein (since we assume biomass consists only of proteins) and the molar weight of glucose $MW_{Glc} = 0.180$ g/mmol glucose. The resulting 'catabolic' yield $Y_{B/S}^{cat}$ predicted by the model is shown by the dashed green line in Fig CC. It exceeds the measured yield at all growth rates. To obtain a more realistic prediction for the yield, we multiply the catabolic yield by the catabolic glucose fraction, giving

$$Y_{B/S}^{cat}(\lambda)\frac{S_{cat}}{S_{tot}} = \frac{v_B}{v_T}\frac{S_{cat}}{S_{tot}} = \frac{B\,h^{-1}}{S_{cat}\,h^{-1}}\frac{S_{cat}}{S_{tot}} = \frac{B}{S_{tot}} = Y_{B/S}^{tot}(\lambda). \quad \text{(S46)}$$

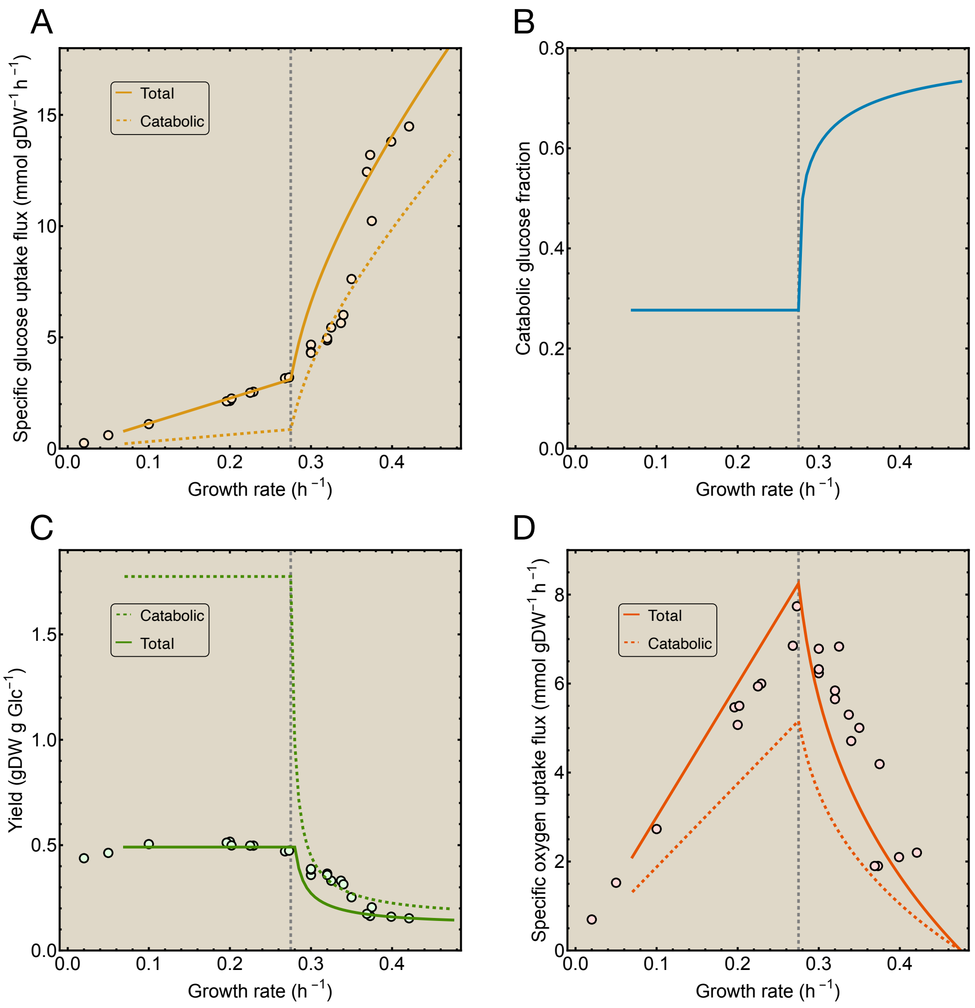

Fig C. **Adjustments for the carbon and oxygen uptake in the yeast model.** A) The total (adjusted) and catabolic specific glucose uptake rate. B) The fraction of glucose consumed by catabolism as function of the growth rate. C) The total (adjusted) and catabolic biomass yield. D) The total (adjusted) and catabolic specific oxygen uptake rate. Chemostat data is reused with permission from [3] (Creative Commons Attribution 4.0 International License).

The solid green line in Fig CC represents the biomass yield per total amount of glucose consumed. This also agrees better with the experimental data.

13 Adjustment and results for oxygen uptake and ATP demand in the yeast model

Respiration requires six moles of oxygen for the complete oxidation of one mole of glucose to carbon dioxide and water. Since the respiratory flux v_R has a unit of moles glucose per hour in the yeast model, the predicted specific oxygen uptake flux is $q_{O_2} = 6\frac{v_R}{\rho_{DW}}$. This is represented by the dashed red line in Fig CD. The model underestimates the oxygen uptake rate compared to the experimental data for two reasons.

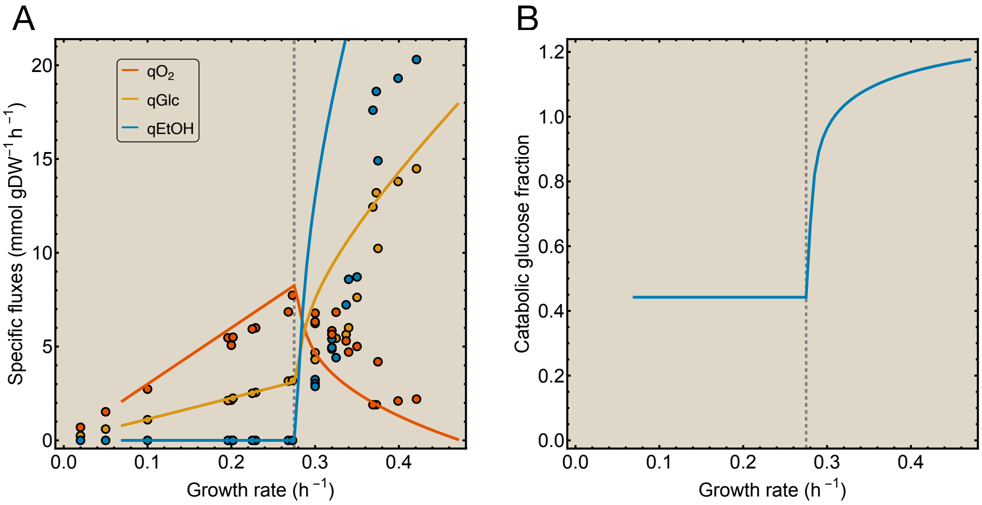

FIG D. **Results of the yeast model accounting for total ATP demand.** A) Specific fluxes predicted by the yeast model together with chemostat data, reused with permission from [3] (Creative Commons Attribution 4.0 International License). B) The fraction of glucose consumed by catabolism as function of the growth rate. This fraction exceeds one when total ATP demand is accounted for.

The first considers the carbon flow through respiration. We assume that all this carbon is converted into carbon dioxide. However, some reactions in the TCA cycle also produce precursors for biosynthesis, like α-ketoglutarate. Taking this into account would increase the respiratory flux, and thus also increase the oxygen uptake rate. However, this would require an assessment of the carbon flow per individual reaction, which is outside the scope of this coarse-grained model.

The second factor that affects the oxygen uptake flux is the ATP demand. Since both glycolysis and the TCA cycle also provide precursors for biosynthesis (such as, respectively, pyruvate and α-ketoglutarate), this makes these pathways in part biosynthetic. Therefore, a fraction of the ATP generated by these pathways is generated during biosynthesis itself. In the core model, we neglect this ATP produced during biosynthesis by subtracting this from the total amount of ATP required per unit biomass. We then only account for the net ATP requirement (m_B) that is provided by one or both of the catabolic modes. Consequently, we regard the respiratory flux in the model as purely catabolic. This results in an underestimation of the predicted specific oxygen uptake flux in comparison to experimental data, since the prediction does not account for oxygen required for ATP production in the TCA cycle during biosynthesis.

To correct for this, we multiply the predicted oxygen uptake flux by $\frac{m_B^{tot}}{m_B}$. This factor represents the total ATP demand (m_B^{tot}) over the net ATP demand. This multiplication gives the increased oxygen uptake required to synthesise this extra ATP. This results in the solid red line in Fig CD, that shows a better correspondence with the experimental data.

13.1 Results of the yeast model for total ATP requirement

Another way to improve the prediction for the oxygen uptake is by increasing the net ATP requirement to the total ATP requirement already before solving the optimisation problem in eq. (S33), instead of adjusting this afterwards. Changing this parameter requires a revised calibration of the yeast model. The Mathematica implementation of this model variant is provided in the source code on Zenodo.

Using the updated calibrated parameters, we again compute the solution to eq. (S33) in terms of the protein fractions. These deviate marginally from the protein fractions found with the net ATP requirement m_B, depicted in Fig 5B. This is because the effect of increasing the ATP demand is (partially) mitigated by the revised calibration.

The results for the specific fluxes are however significantly affected by the increase in ATP demand. A higher ATP demand also requires a higher ATP synthesis flux, i.e., higher glycolytic and respiratory fluxes. An increased respiratory flux results in a higher specific oxygen uptake flux, as represented by the solid red line in Fig DA. As expected, this also agrees better with the experimental data, similar to the adjustment in Fig CD.

An increase in the glycolytic flux also requires a higher glucose uptake rate. This partially accounts for the missing carbon required for biomass synthesis, that we corrected for in the previous section. Nevertheless, this does not account for all missing carbon, so an adjustment like in eq. (S46) is still required. We again plot the adjustment factor, given by the catabolic glucose fraction $\frac{S_{cat}}{S_{tot}}$ in Fig DB. Indeed, this factor is larger compared to Fig CB for growth rates below the critical growth rate, indicating a smaller adjustment. However, after the critical growth rate the catabolic glucose fraction exceeds one. This would suggest that the catabolic glucose requirement predicted by the model exceeds the measured glucose uptake, which includes both catabolism and anabolism. Such a prediction is unrealistic, but follows directly from the higher ATP demand m_B^{tot}. This demand has to be met mostly by ATP generation by glycolysis for growth rates close to λ_{max}. The low glycolytic ATP yield requires an unrealistically high glycolytic flux. This results in a predicted (catabolic) glucose uptake that exceeds the measured glucose uptake. Because this glycolytic flux equals the fermentation flux (during complete fermentation), this also results in an excessive specific ethanol production flux. This is depicted by the blue line in Fig DA, which indeed exceeds the measured ethanol production. In reality, part of the ATP synthesis is still performed by respiration during precursor biosynthesis, thereby reducing both the glycolytic and fermentative flux. However, as mentioned before, taking this into account is outside the scope of this model.

To conclude, using the total ATP requirement results in a better prediction for the oxygen uptake flux, but also in unrealistic predictions for other fluxes. Therefore, we argue that analysing the yeast model with the net ATP requirement and correcting for this afterwards is the better approach. In principle, the core model studies a shift in the catabolic mode and thereby focuses on this part of the metabolic network. This necessarily requires assumptions for the biosynthetic part of the network. These assumptions require adjustments when comparing the model to experimental data.

14 Results for the protein fractions in the yeast model

We process the proteomics data from Elsemman et al. [3] to compare with the model results. First, we account for the special case reactions discussed in Section 10. Then, we categorise the reactions according to the pathways and sectors given in Table A. This is done for all reactions that are assigned to a pathway and for which protein concentrations are measured. Here, we also include measured protein concentrations from batch experiments that were not included to derive the saturation functions in Section 10. We compute the sum of the protein fractions per sector and plot this together with the model predictions as function of the growth rate in Fig 5B.

First, we consider the glycolytic protein fraction. The adjustment to the glucose uptake flux from Section 12 should also be applied to the glycolytic flux, to satisfy the steady-state equations (S28) of the yeast model. Since the mean catalytic constant and saturation of glycolysis are obtained from proteomics data, we can only apply this adjustment by increasing the glycolytic protein fraction. So, we apply the same adjustment to the glycolytic protein fraction in Fig 5B as to the specific glucose uptake flux. Note that the glycolytic protein fraction in Fig 5A is not adjusted, as the protein fractions in the model should add up to one. Despite this adjustment, the glycolytic protein fraction matches the data only qualitatively. This may be due to overestimation of its catalytic constant or saturation function. The glycolytic saturation function vanishes for growth rates below $0.07\,\mathrm{h}^{-1}$, which suggests that it does not accurately represent the mean saturation of glycolysis during slow growth. Because this results in an infeasible optimisation problem, we solve the optimisation problem only for growth rates $\lambda \geq 0.07\,\mathrm{h}^{-1}$. Starting from this value, the glycolytic flux increases with the growth rate. However, the glycolytic protein fraction decreases due to its increasing saturation function, that overcompensates the increased demand in glycolytic flux. Such behaviour is not predicted by the GEM in Elsemman et al. [3], because the authors assume constant saturations.

This behaviour changes after the critical growth rate, because the shift to fermentation requires a higher ATP synthesis rate through glycolysis that can not be compensated by (only) the saturation function.

When the growth rate increases further, the glycolytic protein fraction reaches a roughly constant value. This indicates that the increasing ATP demand is completely met by the increasing saturation function in this regime. All these observations are consistent with the experimental data, except for this last effect.

The predicted respiratory protein fraction matches the data quantitatively and shows the expected decrease after the critical growth rate.

For fermentation, we again include only the two canonical fermentation reactions from the proteomics data. Nevertheless, the model prediction does not fit the data well. This may be explained by preparatory expression of the fermentative enzymes and regulation [16; 17]. Similar to glycolysis, also the predicted fermentative fraction stabilises as the growth rate increases. This also follows from its increasing saturation function.

The biosynthetic protein fraction predicted by the model exceeds the experimental data, likely due to an underestimation of the catalytic constant or the (mean) saturation factor. Furthermore, the predicted biosynthetic protein fraction increases hyperbolically with the growth rate, whereas the data seem to show a linear increase. Such a linear relation between the protein fraction and the growth rate indicates a constant saturation factor. However, this does not agree with the saturation function $f_B(\lambda)$ derived from the same proteomics data.

The remaining protein sectors are the Q-sector, which is assumed to be constant, and the NG-sector. The corresponding protein fractions can not be obtained directly from the proteomics data, since many of these proteins are not assigned to a pathway by Elsemman et al. [3]. Instead, we compute these fractions by subtracting the growth-associated protein concentrations from the total protein concentration. What remains is the growth-unassociated protein, which is the sum of the Q- and the NG-sector. Therefore, we plot these together in Fig 5B. During slow growth (around $\lambda = 0.07\,\mathrm{h}^{-1}$), the model predicts an increasing growth-unassociated protein fraction, due to the rapidly decreasing glycolytic fraction. For higher growth rates, both the data and the prediction for the growth-unassociated fraction decrease. The model prediction reaches its minimum at the critical growth rate. This minimum corresponds to the (constant) Q-sector, which we set to $\phi_Q = 0.25$. In the figure, this is slightly lower due to the adjusted glycolytic protein fraction.

Other factors may also underlie the quantitative discrepancy between the model predictions and the experimental data. Elsemman et al. [3] show that a second metabolic shift occurs in *S. cerevisiae*. Including this results in a better fit of the GEM to the data. This second shift is not contained in our coarse-grained model, as it lacks other proteome sectors that may be constrained. Another effect may arise from the reaction fluxes in terms of averaged kinetic parameters in eq. (31). This approach neglects intrapathway stoichiometry, which also affects the enzyme concentrations.

15 Results of the yeast model for constant saturations

Many models for overflow metabolism include phenomenological concentrations and saturations [14; 15], or set saturation factors constant [3; 4; 18]. Here, we account for changing enzyme saturations by including (mean) saturation functions that change with the growth rate. To test if this extra component results in model predictions that agree better with the experimental data, we also compute the solution to the optimisation problem (S33) for constant saturation factors. We compare the results to the same data.

It is not clear *a priori* what is an appropriate constant value for the saturations. Therefore, we implement two model variants with constant saturations. For the first variant, we evaluate the saturation functions at $\lambda = 0.285\,\mathrm{h}^{-1}$, the median of the growth rate of yeast in the chemostat. For the second case, we set all saturations to one, as also done in other models [3; 4]. Both variants of the model are calibrated again using the methods in Section 11, to find the observed values for the critical and maximal growth rates of yeast.

For the first case, a Mathematica implementation is provided in the source code on Zenodo. Its results are shown in Fig E. Constant saturations result in protein fractions that vary piecewise linear with the growth rate, as is also illustrated by Fig 2A for the general core model. In general, these predicted protein fractions fit the data less well than the prediction obtained with the saturation functions. The predicted glycolytic protein fraction increases monotonically with the growth rate. This is in better agreement with the data after the critical growth rate, but worse before. The predicted respiratory fraction exceeds the data almost four-fold at some growth rates. The biosynthetic fraction increases linearly with the growth rate, however, without an offset on the y-axis. The predictions for the specific fluxes and the biomass yield fit the data

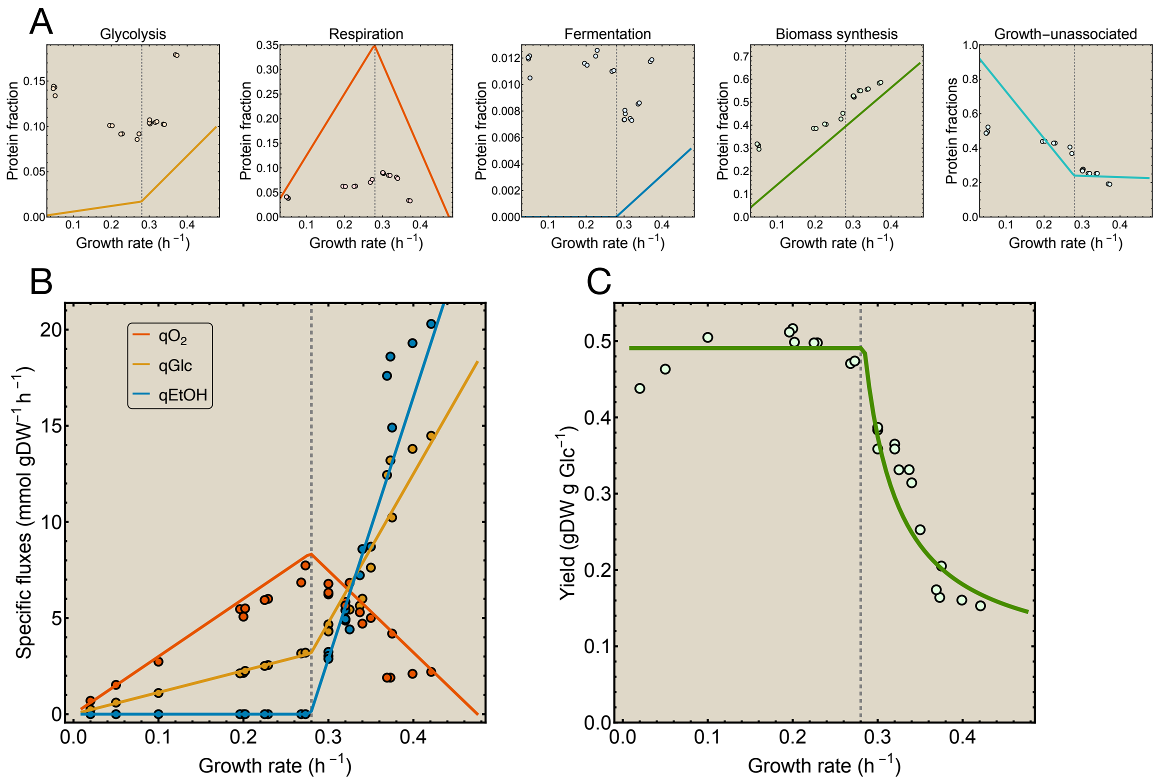

Fig E. **Results of the yeast model for constant saturations factors.** A) Predicted protein fractions for constant saturations together with proteomics data. B) Specific fluxes and C) Biomass yield predicted by the yeast model with constant saturations together with chemostat data. Data is reused with permission from [3] (Creative Commons Attribution 4.0 International License).

slightly better than the predictions for changing saturations. However, this may be misleading, as for both cases the adjustments from Supplementary Sections 12 and 13 are applied. Since the data for specific fluxes show approximately piecewise linear dynamics, piecewise linear predictions appear to be more accurate after applying these adjustments.

The solution of the second model variant shows behaviour that is very similar to the results in Fig E. This follows from the model calibration, where we adjust other parameters to find the observed values for the critical and maximal growth rates. This likely compensates for the effect of increasing the saturations from the value of the saturation functions at $\lambda = 0.285\,\mathrm{h}^{-1}$ to one. Since setting saturations constant gives the model less flexibility, this gives similar results for both cases.

Both cases of constant saturations thereby confirm that accounting for changing saturations leads to a better model prediction.

16 Analytical solution to the yeast model

The metabolic network of the yeast model in Fig 4 also contains two EFMs, respiration and fermentation. Similar to the general core model analysed in Sections 3.1- 3.2, the steady-state equations (S28) of the yeast model have a unique solution if we consider only one of these EFMs as active mode. We express this solution in terms of the protein fractions and the transporter saturation that change as function of the growth rate.

For respiration, we find

$$\begin{aligned}
\phi_B^y(\lambda) &= \frac{N_B}{k_{ribo}\langle f(\lambda)\rangle_B}\lambda \equiv C_B^y(\lambda)\lambda \\
\phi_R^y(\lambda) &= \frac{m_B N_R}{(m_R+m_G)\langle k\rangle_R \langle f(\lambda)\rangle_R}\lambda \equiv C_R^y(\lambda)\lambda \\
\phi_G^{R,y}(\lambda) &= \frac{m_B N_G}{(m_R+m_G)\langle k\rangle_G \langle f(\lambda)\rangle_G}\lambda \equiv C_G^{R,y}(\lambda)\lambda \\
f_T^{R,y}(\lambda) &= \frac{m_B}{(m_R+m_G)\langle k\rangle_T \phi_T^y}\lambda.
\end{aligned} \tag{S47}$$

Here, ϕ_T^y is the constant transporter fraction in the yeast model. The parameter combinations C_j^y, that represent the protein costs per process, now change with the growth rate due to the saturation functions. The corresponding growth-unassociated protein fraction

$$\phi_{NG}^y(\lambda) = 1-(\phi_Q^y+\phi_T^y+\phi_B^y(\lambda)+\phi_R^y(\lambda)+\phi_G^{R,y}(\lambda)) = 1-\phi_Q^y-\phi_T^y-\lambda\left(C_B^y(\lambda)+C_R^y(\lambda)+C_G^{R,y}(\lambda)\right) \tag{S48}$$

again decreases with the growth rate, because e_{tot} is assumed fixed. When this protein pool is depleted, yeast reaches its critical growth rate λ_c^y and shifts to fermentation. However, obtaining an analytical expression for the critical growth rate by solving $\phi_{NG}^y(\lambda)=0$ for λ is not possible, because the protein costs depend on the growth rate. Instead, we find an approximation for λ_c^y by setting the saturation functions constant. Since the observed critical growth rate of yeast during chemostat cultivation is $0.275\,\mathrm{h}^{-1}$, we set the saturation functions constant to their value at $\lambda = 0.275\,\mathrm{h}^{-1}$. This sets the protein costs to $C_j(0.275)\equiv C_j^c$ and gives

$$\lambda_c^y = \frac{1-\phi_Q^y-\phi_T^y}{C_B^c+C_R^c+C_G^{R,c}} \tag{S49}$$

as approximation for the critical growth rate.

For fermentation, the solution to the steady-state equations (S28) is given by

$$\begin{aligned}
\phi_B^y(\lambda) &= \frac{N_B}{k_{ribo}\langle f(\lambda)\rangle_B}\lambda \equiv C_B^y(\lambda)\lambda \\
\phi_F^y(\lambda) &= \frac{m_B N_F}{m_G\langle k\rangle_F \langle f(\lambda)\rangle_F}\lambda \equiv C_F^y(\lambda)\lambda \\
\phi_G^{F,y}(\lambda) &= \frac{m_B N_G}{m_G\langle k\rangle_G \langle f(\lambda)\rangle_G}\lambda \equiv C_G^{F,y}(\lambda)\lambda \\
f_T^{F,y}(\lambda) &= \frac{m_B}{m_G\langle k\rangle_T \phi_T^y}\lambda.
\end{aligned} \tag{S50}$$

Similarly, we obtain an approximate expression for the maximal growth rate λ_{max}^y by evaluating the saturation functions at the observed maximal growth rate, which is $0.47\,\mathrm{h}^{-1}$ for the strain examined in [3]. This sets the protein costs to $C_j(0.47)\equiv C_j^{max}$ and gives

$$\lambda_{max}^y = \frac{1-\phi_Q^y-\phi_T^y}{C_B^{max}+C_F^{max}+C_G^{F,max}}. \tag{S51}$$

The optimisation problem (S33) for the yeast model minimises the transporter saturation f_T. The analytical solutions for the transporter saturation for both modes show that $f_T^{R,y}(\lambda) < f_T^{F,y}(\lambda)$ for all growth rates, because the ATP yield of respiration (m_R+m_G) exceeds that of fermentation (m_G). This confirms respiration as the mode that minimises the glucose uptake rate. Evaluating the transporter saturation of respiration at the critical growth rate and that of fermentation at the maximal growth rate yields

$$\begin{aligned}
f_T^c &\equiv f_T^{R,y}(\lambda_c^y) = \frac{m_B}{(m_R+m_G)\phi_T^y\langle k\rangle_T}\lambda_c^y \\
f_T^{max} &\equiv f_T^{F,y}(\lambda_{max}^y) = \frac{m_B}{m_G\phi_T^y\langle k\rangle_T}\lambda_{max}^y.
\end{aligned} \tag{S52}$$

We combine these equations by computing the ratio of the maximal over the critical transporter saturation, giving

$$\frac{f_T^{max}}{f_T^c} = \frac{m_R + m_G}{m_G} \frac{\lambda_{max}^y}{\lambda_c^y}. \tag{S53}$$

Using the parameters of the yeast model (Table F), we compute the right-hand side of this equation explicitly, which equals 15.4. In reality, the transporter saturation does not deviate that substantially between the critical and the maximal growth rate during chemostat cultivation [3]. However, the coarse-grained structure of our yeast model directly couples these parameters via eq. (S53). This results in values for the transporter saturation that are unrealistically low (around 0.02) at low growth rates.

17 Proteome efficiency in the yeast model

We compute the proteome efficiency of respiration and fermentation in yeast by dividing the ATP synthesis rate by the total protein concentration expended to sustain this ATP flux, similar to the definition in Section 3.3. Using the solutions for the protein fractions for both modes (eq. (S47) and (S50)), this gives

$$\begin{aligned} \epsilon_R^y(\lambda) &= \frac{m_G v_G(\lambda) + m_R v_R(\lambda)}{e_B^y(\lambda) + e_R^y(\lambda) + e_G^{R,y}(\lambda)} = \frac{m_B}{C_B^y(\lambda) + C_R^y(\lambda) + C_G^{R,y}(\lambda)} \\ \epsilon_F^y(\lambda) &= \frac{m_G v_G(\lambda)}{e_B^y(\lambda) + e_F^y(\lambda) + e_G^{F,y}(\lambda)} = \frac{m_B}{C_B^y(\lambda) + C_F^y(\lambda) + C_G^{F,y}(\lambda)}. \end{aligned} \tag{S54}$$

The protein costs related to carbon transport do not appear in the denominator, because the transporter concentration is assumed fixed. Furthermore, the proteome efficiencies in the yeast model depend on the growth rate through the saturation functions. Since these increase monotonically with the growth rate, as demonstrated in Fig B, also the proteome efficiencies increase monotonically with the growth rate. This is depicted in Fig F. Here, the efficiency of fermentation abruptly drops to zero at $\lambda = 0.24$ h^{-1}, because the saturation of fermentation ($f_F(\lambda)$) vanishes at this growth rate. We consider this as a model artifact. The figure shows that the proteome efficiency of fermentation exceeds the proteome efficiency of respiration already before the critical growth rate.

Beyond the critical growth rate, respiration can not be used in isolation because of the extra active constraint. So, to compute the respiratory efficiency also in the $\lambda_c \leq \lambda \leq \lambda_{max}$ regime, we had to neglect the constraint that fixes the total protein concentration e_{tot}. Therefore, we argue that it is better to evaluate and compare the proteome efficiencies only at the critical growth rate $\lambda_c^y = 0.275\,\mathrm{h}^{-1}$. The corresponding values are given in the first line of Table E.

As discussed in Supplementary Section 5, a different definition of the proteome efficiency is given by the ATP synthesis rate per amount of catabolic protein expended. For the yeast model, this gives

$$\begin{aligned} \epsilon_R^{y,cat}(\lambda) &= \frac{m_G v_G(\lambda) + m_R v_R(\lambda)}{e_R^y(\lambda) + e_G^{R,y}(\lambda)} = \frac{m_B}{C_R^y(\lambda) + C_G^{R,y}(\lambda)} \\ \epsilon_F^{y,cat}(\lambda) &= \frac{m_G v_G(\lambda)}{e_F^y(\lambda) + e_G^{F,y}(\lambda)} = \frac{m_B}{C_F^y(\lambda) + C_G^{F,y}(\lambda)}. \end{aligned}$$

Evaluating these at the critical growth rate gives the values in the second line of Table E. This shows that neglecting the biosynthetic protein costs results in much higher values for the proteome efficiency.

These definitions arise from the different interpretations of the protein costs associated with ATP synthesis. For the yeast model, we compute two other values for the proteome efficiency by using the other parameterisation of the ATP requirement for biosynthesis. This is represented by the parameter m_B. In the first two lines of Table E, we computed the proteome efficiencies using the net ATP requirement. The values of the efficiencies using the total ATP requirement for yeast are given in the third and fourth lines. These are computed from the model variant discussed in Supplementary Section 13.

Overall, this results in four different values for the proteome efficiency of both modes in the yeast model. Despite their significant variation, they all meet the condition that fermentation is more proteome-efficient than respiration. Therefore, all these definitions are interchangeable. What matters here are the principles:

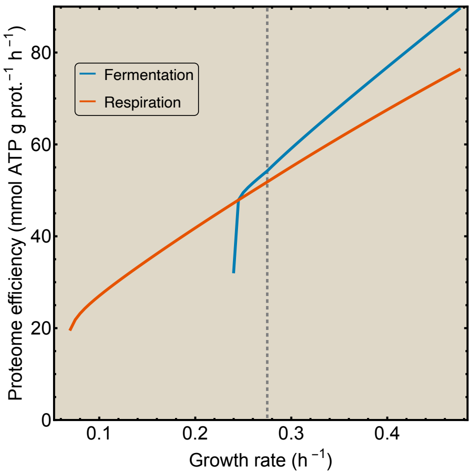

FIG F. **Proteome efficiencies of both metabolic modes in yeast.** Proteome efficiencies for respiration and fermentation computed as the ATP synthesis rate per total protein concentration expended to sustain the ATP flux. They change as function of the growth rate due to changing saturations. The dashed line represents the critical growth rate.

a shift from respiration towards fermentation due to a limited total protein concentration implies a higher proteome efficiency of fermentation.

The literature values for the proteome efficiency also differ significantly, even when obtained for the same organism. This holds for both *E. coli* [4; 18; 19] and *S. cerevisiae* [3; 6; 18; 19]. In the case of Shen et al. [6], the authors even estimated a higher proteome efficiency for respiration than for fermentation, which violates the condition (S12). Indeed, if overflow metabolism is caused by a limitation on the total proteome, this can not be true. We conjecture that their finding results from an alternative estimate of the protein costs associated with respiration and fermentation. For instance, if protein costs for synthesis of mitochondria are neglected, this results in an increased proteome efficiency for respiration.

**Definition of proteome efficiency**	**Proteome efficiency respiration** (mmol ATP gram protein^{-1} h^{-1})	**Proteome efficiency fermentation** (mmol ATP gram protein^{-1} h^{-1})
Net ATP synthesis rate per total growth-associated protein	51.8	54.2
Net ATP synthesis rate per catabolic protein	346	492
Total ATP synthesis rate per total growth-associated protein	83	84.4
Total ATP synthesis rate per catabolic protein	483	533

TABLE E. **Table with different definitions of the proteome efficiency.** For each definition, the proteome efficiencies for respiration and fermentation in yeast are computed at the critical growth rate $\lambda_c^y = 0.275\,\mathrm{h}^{-1}$. For all cases, fermentation has a higher proteome efficiency than respiration. The values for the proteome efficiencies are computed using the Mathematica implementations of the different yeast model variants, provided in the source code on Zenodo.

18 Break-even analysis for the yeast model

Here, we apply the break-even analysis in terms of parameter perturbations of Section 3.5 and Section 6 to the yeast model. To do this, we should compute λ_{ratio}, the ratio of the maximal growth rate (eq. (S51)) over the critical growth rate (eq. (S49)). However, because these growth rates are computed at different saturation values, this is no fair comparison of the protein costs. Therefore, we evaluate the saturation functions at $\lambda = 0.37$ hr^{-1}, the mean of the critical and the maximal growth rate. The break-even condition then becomes

$$\lambda_{ratio} = \frac{C_B(0.37) + C_R(0.37) + C_G^R(0.37)}{C_B(0.37) + C_F(0.37) + C_G^F(0.37)} = 1. \tag{S55}$$

We reformulate this condition in terms of the model parameters as

$$\frac{N_R}{(m_R + m_G)\langle k\rangle_R \langle f(0.37)\rangle_R} = \frac{N_F}{m_G \langle k\rangle_F \langle f(0.37)\rangle_F} + \frac{m_R N_G}{m_G(m_R + m_G)\langle k\rangle_G \langle f(0.37)\rangle_G}. \tag{S56}$$

We study this condition for five parameter pairs in the yeast model. These correspond to the parameter pairs from the core model that are used to obtain the phase diagrams in Fig 3. For the yeast model, we use its Mathematica implementation given in the source code on Zenodo, which results in the phase diagrams shown in Fig G. Figs GA, B and E depict the results for parameters that appear in both the general and the yeast model. These agree with the results for the general core model, respectively in Figs 3B, C and F.

Figs GC and D contain the results for perturbation of glycolytic parameters (k_G and m_G). Since glycolysis is used by both modes, we expect it to affect both respiration and fermentation. Therefore, we expect results different from those obtained for perturbation of the corresponding fermentative parameters (k_F and m_F) in the general core model (see Fig 3D and E). However, also in these cases, the results of both models show qualitative agreement. This indicates that perturbing glycolysis mainly affects fermentation.

An advantage over the phase diagrams in Fig 3 is that we can add a coordinate in Fig G that represents the (calibrated) parameter configuration in the yeast model. For *S. cerevisiae*, this coordinate always falls within the blue region, that represents the regime in which a metabolic shift occurs. The distance of this coordinate with respect to the curve (representing the break-even condition (S56)) gives an estimation for the perturbation required to remove overflow metabolism in yeast strains, as a shift will not occur when the coordinate enters the brown region. Parameter pairs for which this distance is (relatively) small may be useful biotechnological targets to manipulate the metabolic shift.

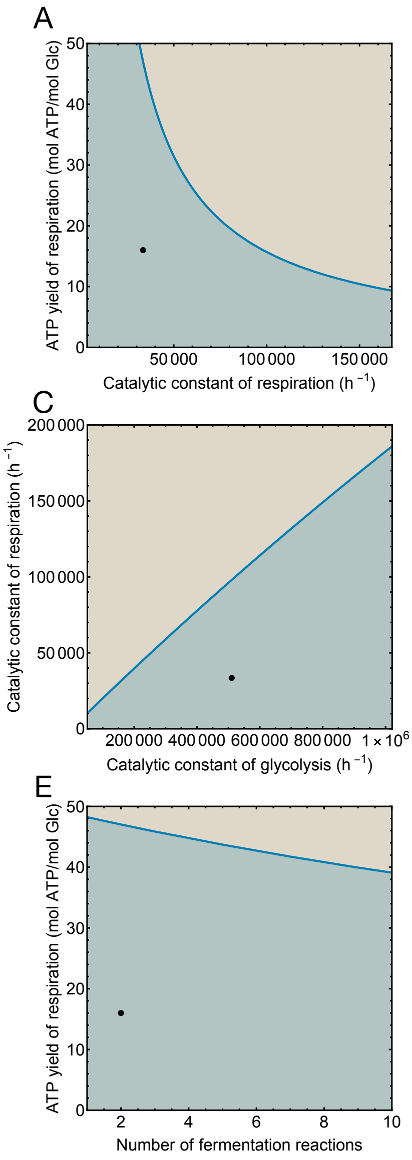

Fig G. **Phase diagrams for the yeast model.** A)-E) The break-even analysis applied to the yeast model in terms of several model parameter pairs. For parameter configurations in the blue region, a metabolic shift occurs. In the brown region a shift will never occur. The black dot represents the parameter configuration used in the yeast model.

Parameter symbol	Parameter description	Value (source)
MW_e	Average molecular weight of protein	50 g/mmol protein ([20])
N_e	Average number of proteins	$2.5 \cdot 10^6$ proteins/μm^3 ([20])
e_{tot}	Average total protein concentration	4.15 mmol protein/L cells (convert N_e)
ϕ_T^y	Transporter fraction of the total proteome	0.3% (estimated from proteomics data [3])
ϕ_Q^y	Q-sector fraction of the total proteome	25% (estimated from proteomics data [3])
ρ_e	Protein density	$MW_e e_{tot} = 208$ g/L
$\eta_{e/DW}$	Protein mass fraction per gram dry weight	40 % ([20])
ρ_{DW}	Dry weight density	$\frac{\rho_e}{\eta_{e/DW}} = 519$ gDW/L
$Y_{ATP/gDW}$	Net ATP requirement per gram dry weight	56.3 mmol ATP/gDW ([10])
m_B	Net ATP requirement per mmol protein	$\frac{Y_{ATP/gDW}\rho_{DW}}{e_{tot}} = 7038$ mmol ATP/mmol protein
$Y_{ATP/gDW}^{tot}$	Total ATP requirement per gram dry weight	90 mmol ATP/gDW ([10])
m_B^{tot}	Total ATP requirement per mmol protein	$\frac{Y_{ATP/gDW}^{tot}\rho_{DW}}{e_{tot}} = 11250$ mmol ATP/mmol protein
m_G	ATP yield of glycolysis	2 mmol ATP/mmol glucose
m_R	ATP yield of respiration (Oxidative phosphorylation and TCA cycle)	16 mmol ATP/mmol glucose
$Y_{O_2/Glc}$	Oxygen demand per glucose during respiration	6 mol O_2/mol glucose
$Y_{Eth/Glc}$	Ethanol yield per glucose during fermentation	2 mol ethanol/mol glucose
k_{ribo}	Catalytic constant of the ribosome	126 h^{-1} ([3])

Table F. **Parameters used in the yeast model.** All parameter values are obtained from the literature or computed using literature values.

References

[1] Sheng Hui, Josh M Silverman, Stephen S Chen, David W Erickson, Markus Basan, Jilong Wang, Terence Hwa, and James R Williamson. Quantitative proteomic analysis reveals a simple strategy of global resource allocation in bacteria. *Molecular systems biology*, 11(2):784, 2015.

[2] Nathan M Belliveau, Griffin Chure, Christina L Hueschen, Hernan G Garcia, Jane Kondev, Daniel S Fisher, Julie A Theriot, and Rob Phillips. Fundamental limits on the rate of bacterial growth and their influence on proteomic composition. *Cell systems*, 12(9):924–944, 2021.

[3] Ibrahim E Elsemman, Angelica Rodriguez Prado, Pranas Grigaitis, Manuel Garcia Albornoz, Victoria Harman, Stephen W Holman, Johan van Heerden, Frank J Bruggeman, Mark MM Bisschops, Nikolaus Sonnenschein, et al. Whole-cell modeling in yeast predicts compartment-specific proteome constraints that drive metabolic strategies. *Nature communications*, 13(1):801, 2022.

[4] Markus Basan, Sheng Hui, Hiroyuki Okano, Zhongge Zhang, Yang Shen, James R. Williamson, and Terence Hwa. Overflow metabolism in *Escherichia coli* results from efficient proteome allocation. *Nature*, 528(7580):99–104, December 2015. doi: 10.1038/nature15765. URL `https://doi.org/10.1038/nature15765`. ISBN: 1476-4687.

[5] Julius Battjes, Pranas Grigaitis, Milou Hoving, Thomas Visser, Karina Stampfl, Gabriela Miguel, Johan H Van Heerden, Jesper Andersen, Frank Bruggeman, and Bas Teusink. Mitochondrial efficiency determines Crabtree effect across yeasts, November 2024. URL `http://biorxiv.org/lookup/doi/10.1101/2024.11.01.621473`.

[6] Yihui Shen, Hoang V. Dinh, Edward R. Cruz, Zihong Chen, Caroline R. Bartman, Tianxia Xiao, Catherine M. Call, Rolf-Peter Ryseck, Jimmy Pratas, Daniel Weilandt, Heide Baron, Arjuna Subramanian, Zia Fatma, Zong-Yen Wu, Sudharsan Dwaraknath, John I. Hendry, Vinh G. Tran, Lifeng Yang, Yasuo Yoshikuni, Huimin Zhao, Costas D. Maranas, Martin Wühr, and Joshua D. Rabinowitz. Mitochondrial ATP generation is more proteome efficient than glycolysis. *Nature Chemical Biology*, March 2024. ISSN 1552-4450, 1552-4469. doi: 10.1038/s41589-024-01571-y. URL `https://www.nature.com/articles/s41589-024-01571-y`.

[7] Birgit Dabrock, Hubert Bahl, and Gerhard Gottschalk. Parameters affecting solvent production by *Clostridium pasteurianum*. *Applied and Environmental Microbiology*, 58(4):1233–1239, 1992. doi: 10.1128/aem.58.4.1233-1239.1992. URL `https://journals.asm.org/doi/abs/10.1128/aem.58.4.1233-1239.1992`.

[8] Matthew Scott, Carl W. Gunderson, Eduard M. Mateescu, Zhongge Zhang, and Terence Hwa. Interdependence of cell growth and gene expression: Origins and consequences. *Science*, 330(6007):1099–1102, 2010. doi: 10.1126/science.1192588. URL `https://www.science.org/doi/abs/10.1126/science.1192588`.

[9] Avik Mukherjee, Yu-Fang Chang, Yanqing Huang, Nina Catherine Benites, Leander Ammar, Jade Ealy, Mark Polk, and Markus Basan. Plasticity of growth laws tunes resource allocation strategies in bacteria. *PLOS Computational Biology*, 20(1):1–19, 01 2024.

[10] Maaike Remeijer and Frank Bruggeman. Entangled stoichiometric objectives shape microbial catabolism. *bioRxiv*, 2025. doi: 10.1101/2025.07.29.667445. URL `https://www.biorxiv.org/content/early/2025/07/30/2025.07.29.667445`.

[11] Jianye Xia, Benjamin J. Sánchez, Yu Chen, Kate Campbell, Sergo Kasvandik, and Jens Nielsen. Proteome allocations change linearly with the specific growth rate of *Saccharomyces cerevisiae* under glucose limitation. *Nature Communications*, 13(1):2819, May 2022. ISSN 2041-1723. doi: 10.1038/s41467-022-30513-2. URL `https://www.nature.com/articles/s41467-022-30513-2`.

[12] Philipp Erdrich, Ralf Steuer, and Steffen Klamt. An algorithm for the reduction of genome-scale metabolic network models to meaningful core models. *BMC Systems Biology*, 9(1):48, August 2015. ISSN 1752-0509. doi: 10.1186/s12918-015-0191-x. URL `https://doi.org/10.1186/s12918-015-0191-x`.

[13] Michael Y Galperin, Roberto Vera Alvarez, Svetlana Karamycheva, Kira S Makarova, Yuri I Wolf, David Landsman, and Eugene V Koonin. COG database update 2024. *Nucleic Acids Research*, 53(D1): D356–D363, November 2024. ISSN 1362-4962. doi: 10.1093/nar/gkae983. URL `https://doi.org/10.1093/nar/gkae983`.

[14] Douwe Molenaar, Rogier Van Berlo, Dick De Ridder, and Bas Teusink. Shifts in growth strategies reflect tradeoffs in cellular economics. *Molecular Systems Biology*, 5(1):323, January 2009. ISSN 1744-4292, 1744-4292. doi: 10.1038/msb.2009.82. URL `https://www.embopress.org/doi/10.1038/msb.2009.82`.

[15] Daan H. De Groot, Coco Van Boxtel, Robert Planqué, Frank J. Bruggeman, and Bas Teusink. The number of active metabolic pathways is bounded by the number of cellular constraints at maximal metabolic rates. *PLOS Computational Biology*, 15(3):e1006858, March 2019. ISSN 1553-7358. doi: 10.1371/journal.pcbi.1006858. URL `https://dx.plos.org/10.1371/journal.pcbi.1006858`.

[16] Jack T. Pronk, Yde H. Steensma, and Johannes P. van Dijken. Pyruvate metabolism in *Saccharomyces cerevisiae*. *Yeast*, 12(16):1607–1633, 1996. doi: https://doi.org/10.1002/(SICI)1097-0061(199612)12:16<1607::AID-YEA70>3.0.CO;2-4. URL `https://onlinelibrary.wiley.com/doi/abs/10.1002/%28SICI%291097-0061%28199612%2912%3A16%3C1607%3A%3AAID-YEA70%3E3.0.CO%3B2-4`.

[17] Leandro José de Assis, Russolina Benedeta Zingali, Claudio Akio Masuda, Silas Pessini Rodrigues, and Monica Montero-Lomelí. Pyruvate decarboxylase activity is regulated by the Ser/Thr protein phosphatase Sit4p in the yeast *Saccharomyces cerevisiae*. *FEMS Yeast Research*, 13(6):518–528, September 2013. ISSN 1567-1356. doi: 10.1111/1567-1364.12052. URL `https://doi.org/10.1111/1567-1364.12052`.

[18] Yu Chen and Jens Nielsen. Energy metabolism controls phenotypes by protein efficiency and allocation. *Proceedings of the National Academy of Sciences*, 116(35):17592–17597, August 2019. ISSN 0027-8424, 1091-6490. doi: 10.1073/pnas.1906569116. URL `https://pnas.org/doi/full/10.1073/pnas.1906569116`.

[19] Xin Wang. Overflow metabolism originates from growth optimization and cell heterogeneity. *eLife*, 13:RP94586, June 2025. ISSN 2050-084X. doi: 10.7554/eLife.94586.4. URL `https://elifesciences.org/articles/94586`.

[20] Ron Milo. What is the total number of protein molecules per cell volume? A call to rethink some published values. *BioEssays*, 35(12):1050–1055, 2013. doi: https://doi.org/10.1002/bies.201300066. URL `https://onlinelibrary.wiley.com/doi/abs/10.1002/bies.201300066`.
